# Selective Targeting of Human and Animal Pathogens of the *Helicobacter* Genus by Flavodoxin Inhibitors: Efficacy, Synergy, Resistance and Mechanistic Studies

**DOI:** 10.3390/ijms221810137

**Published:** 2021-09-20

**Authors:** Sandra Salillas, Juan José Galano-Frutos, Alejandro Mahía, Ritwik Maity, María Conde-Giménez, Ernesto Anoz-Carbonell, Helena Berlamont, Adrian Velazquez-Campoy, Eliette Touati, Uwe Mamat, Ulrich E. Schaible, José A. Gálvez, María D. Díaz-de-Villegas, Freddy Haesebrouck, José A. Aínsa, Javier Sancho

**Affiliations:** 1 Biocomputation and Complex Systems Physics Institute (BIFI)-Joint Units: BIFI-IQFR (CSIC) and GBsC-CSIC, University of Zaragoza, 50018 Zaragoza, Spain; sandrasalillasberges@gmail.com (S.S.); juanjogf@gmail.com (J.J.G.-F.); amahia@unizar.es (A.M.); mr.ritwikmaity@outlook.com (R.M.); mcondeg@unizar.es (M.C.-G.); eanoz@unizar.es (E.A.-C.); adrianvc@unizar.es (A.V.-C.); ainsa@unizar.es (J.A.A.); 2 Departamento de Bioquímica y Biología Molecular y Celular, Faculty of Science, University of Zaragoza, 50009 Zaragoza, Spain; 3 Aragon Health Research Institute (IIS Aragón), 50009 Zaragoza, Spain; 4 Departamento de Microbiología, Pediatría, Radiología y Salud Pública, Faculty of Medicine, University of Zaragoza, 50009 Zaragoza, Spain; 5 Department of Pathobiology, Pharmacology and Zoological Medicine, Faculty of Veterinary Medicine, Ghent University, Salisburylaan 133, B9820 Merelbeke, Belgium; Helena.Berlamont@UGent.be (H.B.); freddy.haesebrouck@ugent.be (F.H.); 6 ARAID Foundation, Government of Aragon, 50018 Zaragoza, Spain; 7 CIBER de Enfermedades Hepáticas y Digestivas CIBERehd, Instituto de Salud Carlos III, 28029 Madrid, Spain; 8 Unit of Helicobacter Pathogenesis, CNRS UMR2001, Department of Microbiology, Institut Pasteur, 25-28 Rue du Dr. Roux, 75724 Paris, France; eliette.touati@pasteur.fr; 9 Cellular Microbiology, Program Area Infections, Research Center Borstel, Leibniz Lung Center, 23845 Borstel, Germany; umamat@fz-borstel.de (U.M.); uschaible@fz-borstel.de (U.E.S.); 10 Instituto de Síntesis Química y Catálisis Homogénea (ISQCH), CSIC—Departamento de Química Orgánica, Faculty of Science, University of Zaragoza, 50009 Zaragoza, Spain; jagl@unizar.es (J.A.G.); loladiaz@unizar.es (M.D.D.-d.-V.); 11 CIBER de Enfermedades Respiratorias—CIBERES, Instituto de Salud Carlos III, 28029 Madrid, Spain

**Keywords:** *Helicobacter*, narrow-spectrum antimicrobial, AMR, flavodoxin, drug discovery

## Abstract

Antimicrobial resistant (AMR) bacteria constitute a global health concern. *Helicobacter pylori* is a Gram-negative bacterium that infects about half of the human population and is a major cause of peptic ulcer disease and gastric cancer. Increasing resistance to triple and quadruple *H. pylori* eradication therapies poses great challenges and urges the development of novel, ideally narrow spectrum, antimicrobials targeting *H. pylori*. Here, we describe the antimicrobial spectrum of a family of nitrobenzoxadiazol-based antimicrobials initially discovered as inhibitors of flavodoxin: an essential *H. pylori* protein. Two groups of inhibitors are described. One group is formed by narrow-spectrum compounds, highly specific for *H. pylori*, but ineffective against enterohepatic *Helicobacter* species and other Gram-negative or Gram-positive bacteria. The second group includes extended-spectrum antimicrobials additionally targeting Gram-positive bacteria, the Gram-negative *Campylobacter jejuni*, and most *Helicobacter* species, but not affecting other Gram-negative pathogens. To identify the binding site of the inhibitors in the flavodoxin structure, several *H. pylori*-flavodoxin variants have been engineered and tested using isothermal titration calorimetry. An initial study of the inhibitors capacity to generate resistances and of their synergism with antimicrobials commonly used in *H. pylori* eradication therapies is described. The narrow-spectrum inhibitors, which are expected to affect the microbiota less dramatically than current antimicrobial drugs, offer an opportunity to develop new and specific *H. pylori* eradication combinations to deal with AMR in *H. pylori*. On the other hand, the extended-spectrum inhibitors constitute a new family of promising antimicrobials, with a potential use against AMR Gram-positive bacterial pathogens.

## 1. Introduction

*Helicobacter pylori* (*H. pylori*) is a Gram-negative proteobacterium estimated to infect about 50% of the human population worldwide [1]. Although *H. pylori* infection remains often asymptomatic [2], it is an important cause of peptic ulcer disease, MALT lymphoma, and gastric cancer [3,4], and *H. pylori* is the only bacterial pathogen considered as a Class I carcinogen [5]. *H. pylori* eradication is carried out using either triple or quadruple chemotherapy, in which several antibiotics and antimicrobial compounds including bismuth are combined with a proton pump inhibitor (PPI) [6]. The increasing development of resistance to antimicrobials used in *H. pylori* eradication therapies has led to the inclusion of clarithromycin (Cla)-resistant *H. pylori* as a Priority 2 pathogen in the WHO global priority pathogens list, urging the development of novel antimicrobials to treat the infection [7]. On the other hand, no specific or highly selective *H. pylori* antimicrobial exists, which prevents the design of precision eradication therapies that could minimise both the impact on the patients’ microbiota and the multiplication of resistances due to the massive use of broad-spectrum antimicrobials [8]. 

**Figure 1:**
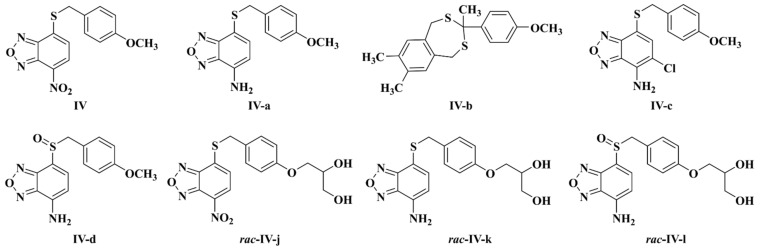
Compounds tested for antimicrobial activity against bacteria.

To meet the challenges represented by *H. pylori* eradication, a variety of new targets for pharmacological intervention are being studied [9] including *H. pylori* flavodoxin (*Hp*-Fld) [10]. Flavodoxins are bacterial electron carriers, not present in humans, that participate in different redox reactions and that, depending on the bacteria, may be essential or non-essential proteins [10,11]. *Hp*-Fld [12,13] is an essential protein that mediates the oxidative decarboxylation of pyruvate by pyruvate-oxidoreductase [14]. It belongs to the long-chain flavodoxin class, differing from short-chain flavodoxins by the presence of an extra loop that may play a role in the binding to partner proteins [15]. While all bacterial flavodoxins, either long or short-chain, are quite similar at the structural level, *Hp*-Fld contains a distinct pocket near the binding site of the FMN (flavin mononucleotide) redox cofactor [12]. As the binding of small molecules at such a pocket might serve to inhibit electron transfer by the FMN cofactor or to impair the binding of partner proteins to *Hp*-Fld [11], a high throughput screening was run to identify flavodoxin binders from a diverse chemical library of 10,000 compounds [16]. Furthermore, by using an in vitro coupled reaction, several binders proved to inhibit *Hp*-Fld activity and, interestingly, they were subsequently shown to be either bactericidal (three of them) or bacteriostatic for *H. pylori*. After several rounds of chemical variation and efficacy testing [17,18], a family of novel nitrobenzoxadiazol-based antimicrobials has emerged, led by compound **IV** (Figure 1) [18]. These antimicrobials have been provisionally described as potentially specific for *H. pylori*, but a detailed analysis of their spectrum of antibacterial activity is lacking. On the other hand, these compounds have been tested in a mouse model of *H. pylori* infection as a monotherapy only [18]. Therefore, further knowledge of the potential synergy of these compounds with compounds currently used in triple/quadruple anti-*H. pylori* therapies (such as Cla, metronidazole (Mnz), or PPIs) could serve to investigate the efficacy of such novel combination therapies in mice, with the aim of proposing new combination therapies to treat *H. pylori* infections in humans.

In this work, we describe the antimicrobial spectrum of lead compound **IV** and variants thereof for bacteria belonging to 15 bacterial genera (7 Gram-negatives and 8 Gram-positives), including a more detailed characterization of their activity against 9 species of the *Helicobacter* genus (*H. pylori* plus 8 non-*H. pylori Helicobacter* species: NHPH) capable of infecting humans and/or domestic animals and having been described as causative agents of gastric or hepatic pathologies [19,20]. Besides, we obtain valuable mechanistic information concerning the binding of the *Hp*-Fld inhibitors to the target protein and their potential capacity to generate new resistances. As it appears, aniline-bearing **IV** derivatives are narrow-spectrum antimicrobials specific for *H. pylori* (and possibly for other gastric *Helicobacter* species), while those bearing a nitro group are extended-spectrum antimicrobials, which are also effective against most Gram-positive bacteria tested.

## 2. Materials and Methods

### 2.1. Reagents and Chemicals

Compounds **IV**, **IV-a**, **IV-b**, **IV-c**, **IV-d** (Figure 1) were previously synthesised as described and their MCC_50_. for eukaryotic cells were determined [18]. The synthesis of compounds ***rac*-IV-j**, ***rac*-IV-k** and ***rac*-IV-l** is described below. Their MCC_50_. for eukaryotic cells follow the trends of the respective parent compounds (not shown). The antibiotics metronidazole (**Mnz**) and clarithromycin (**Cla**) and the proton pump inhibitors (PPIs) omeprazole and rabeprazole were purchased from Acros Organics, Sigma-Aldrich, Fluorochem and Tokyo Chemical Industry, respectively, whereas the bacterial efflux inhibitors (EIs) carbonyl cyanide 3-chlorophenylhydrazone (CCCP), reserpine and valinomycin were obtained from Sigma-Aldrich. All chemicals were dissolved in 100% dimethylsulfoxide (DMSO) and stored frozen at −20 °C. Resazurin sodium salt solution was prepared at 0.01% (*w*/*v*) in distilled water, sterilised by filtering and stored at 4 °C for up to two weeks. Flavin mononucleotide (FMN) was acquired from Santa Cruz Biotechnology.

### 2.2. Synthesis of Compounds **Rac-*IV-j***, **Rac-*IV-k*** and **Rac-*IV-l***

**Figure 2:**
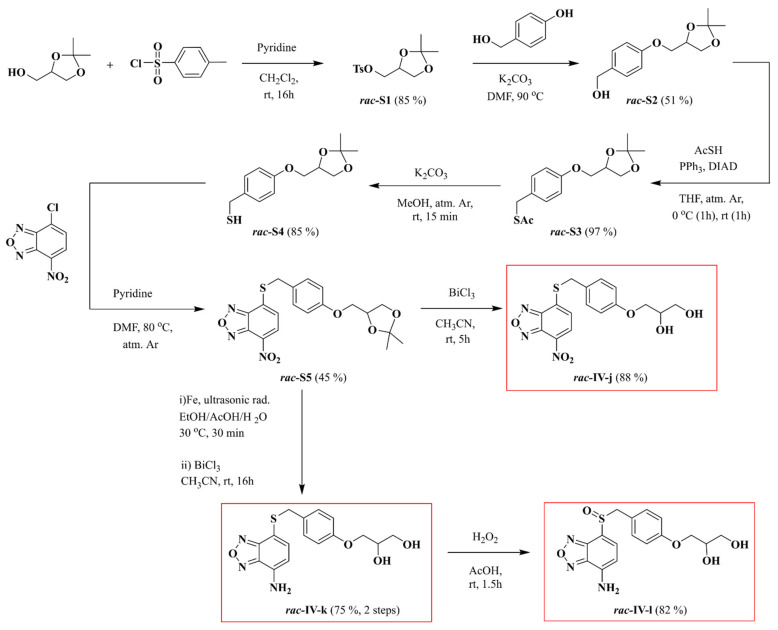
Scheme of the synthesis of compounds ***rac*-IV-j**, ***rac*-IV-k** and ***rac*-IV-l**.

Unless otherwise specified, all reagents for synthesis were obtained from commercial suppliers and were used without purification. TLC was performed on precoated silica gel polyester plates, and products were visualised using UV light (254 nm) and ninhydrin, anisaldehyde, or potassium permanganate solutions, followed by heating. Column chromatography was performed on silica gel 60 (70–200 µm) with air pressure. Melting points were determined in open glass capillaries with a Gallenkamp apparatus. Infrared spectra were recorded with a Fourier transform infrared spectrometer (Thermo Nicolet Avatar 360 FT-IR (Thermo-Fischer Scientific, Waltham, MA, USA)). NMR spectra were recorded with a Bruker AV-400 spectrometer (Bruker-Biospin, Rheinstetten, Germany) (400 MHz for ^1^H-NMR experiments and 100 MHz for ^13^C-NMR) or a Bruker AV-300 spectrometer (Bruker-Biospin, Rheinstetten, Germany) (300 MHz for ^1^H-NMR experiments) in the stated deuterated solvents. ^1^H and ^13^C chemical shifts were referenced to internal solvent resonances and reported in ppm relative to tetramethylsilane. *J* values are given in Hz. High-resolution positive (or negative) electrospray ionisation mass spectra were recorded with a Bruker Daltonics MicroToF-Q spectrometer (BrukerDaltonics, Billerica, MA, USA) with use of ultradilute solutions of the chemical compounds in methanol. A scheme of the synthesis of compounds ***rac*-IV-j**, ***rac*-IV-k** and ***rac*-IV-l** is shown in Figure 2.

#### 2.2.1. ***rac-(2,2-Dimethyl-1,3-dioxolan-4-yl)methyl p-Toluenenesulfonate (rac-S1)***

A suspension of p-toluenesulfonyl chloride (1.51 g, 7.92 mmol) in anhydrous dichloromethane (3.0 mL) was added dropwise to a solution of (2,2-dimethyl-1,3-dioxolan-4-yl)methanol (872 mg, 6.60 mmol) and pyridine (1.6 mL, 19.8 mmol) in anhydrous dichloromethane (4.0 mL) (Figure 2). The reaction mixture was stirred at room temperature for 16 h. Then, the reaction mixture was washed with water (10 mL) and the aqueous phase was extracted with dichloromethane (10 mL). The combined organic layers were dried with anhydrous MgSO_4_, filtered and evaporated under reduced pressure. The resulting residue was purified by column chromatography (eluent: Et_2_O/hexane 1:1) to afford compound ***rac*-S1** (1.60 g, 85% yield) as a white solid. **mp**: 48–49 °C. **IR** (KBr, ν_max_/cm^−1^): 1169, 1347, 1457, 1495, 1595. **^1^H-NMR** (300 MHz, CDCl_3_, δ): 7.82–7.74 (m, 2H), 7.38–7.31 (m, 2H), 4.31–4.22 (m, 1H), 4.06–3.92 (m, 3H), 3.75 (dd, 1H, J = 9.0, J = 5.1), 2.44 (s, 3H), 1.33 (s, 3H), 1.30 (s, 3H). **^13^C-NMR** (100 MHz, CDCl_3_, δ): 145.0, 132.6, 129.9, 127.9, 110.0, 72.9, 69.4, 66.1, 26.6, 25.1, 21.6. **HRMS (ESI^+^)**: *m*/*z* [M + Na]^+^ calculated for C_13_H_18_NaO_5_S 309.0768, found 309.0754.

#### 2.2.2. ***rac-(4-((2,2-Dimethyl-1,3-dioxolan-4-yl)methoxy)phenyl)methanol (rac-S2)***

To a solution of compound ***rac*-S1** (802 mg, 2.80 mmol) and 4-hydroxybenzyl alcohol (417 mg, 3.36 mmol) in anhydrous dimethylformamide (6.0 mL) was added dry K_2_CO_3_ (464 mg, 3.36 mmol) and the resulting suspension was stirred for 16 h at 90 °C. The solvent was then removed under reduced pressure and the resulting residue was dissolved in distilled water (15 mL) and extracted with dichloromethane (2 × 10 mL). The combined organic layers were dried with anhydrous MgSO_4_, filtered and evaporated under reduced pressure. The resulting residue was purified by column chromatography (eluent: Et_2_O/hexane 1:1) to afford compound ***rac*-S2** (339 mg, 51% yield) as a white solid. **mp**: 47–48 °C. **IR** (KBr, ν_max_/cm^−1^): 1453, 1514, 1614, 3402. **^1^H-NMR** (300 MHz, CDCl_3_, δ): 7.32–7.25 (m, 2H), 6.93–6.87 (m, 2H), 4.61 (s, 2H), 4.53–4.43 (m, 1H), 4.17 (dd, 1H, J = 8.4, J = 6.3), 4.06 (dd, 1H, J = 9.6, J = 5.4), 3.94 (dd, 1H, J = 9.6, J = 6.0), 3.90 (dd, 1H, J = 8.4, J = 5.7), 1.83 (sa, 1H), 1.47 (s, 3H), 1.41 (s, 3H). **^13^C-NMR** (100 MHz, CDCl_3_, δ): 158.1, 133.6, 128.5, 114.5, 109.7, 73.9, 68.8, 66.8, 64.8, 26.7, 25.3. **HRMS (ESI^+^)**: *m*/*z* [M + Na]^+^ calculated for C_13_H_18_NaO_4_ 261.1098, found 261.1093.

#### 2.2.3. ***rac-S-(4-((2,2-Dimethyl-1,3-dioxolan-4-yl)methoxy)benzyl) Thioacetate (rac-S3)***

To a cooled (0 °C) solution of PPh_3_ (671 mg, 2.56 mmol) in anhydrous THF (8.0 mL) was added diisopropyl azodicarboxylate (0.50 mL, 2.56 mmol) and the resulting suspension was stirred for 20 min at 0 °C under an argon atmosphere. Then thioacetic acid (0.18 mL, 2.56 mmol) and a solution of compound ***rac*-S2** (305 mg, 1.28 mmol) in anhydrous THF (16 mL) were consecutively added. The reaction mixture was stirred under an argon atmosphere for 1 h at 0 °C and for 1 additional hour at room temperature. The solvent was removed under reduced pressure and the resulting residue was purified by column chromatography (eluent 1: hexane/Et_2_O 9:1, eluent 2: hexane/Et_2_O 8:2) to afford compound ***rac*-S3** (368 mg, 97% yield) as a white solid. **mp**: 57–58 °C. **IR** (KBr, ν_max_/cm^−1^): 1455, 1514, 1612, 1687. **^1^H-NMR** (300 MHz, CDCl_3_, δ): 7.23–7.16 (m, 2H), 6.87–6.80 (m, 2H), 4.51–4.40 (m, 1H), 4.15 (dd, 1H, J = 8.4, J = 6.6), 4.07 (s, 2H), 4.03 (dd, 1H, J = 9.6, J = 5.4), 3.96–3.83 (m, 2H), 2.33 (s, 3H), 1.45 (s, 3H), 1.40 (s, 3H). **^13^C-NMR** (100 MHz, CDCl_3_, δ): 195.2, 157.7, 130.1, 129.9, 114.6, 109.7, 73.9, 68.8, 66.8, 32.8, 30.3, 26.7, 25.3. **HRMS (ESI^+^)**: *m*/*z* [M + Na]^+^ calculated for C_15_H_20_NaO_4_S 319.0975, found 319.0983.

#### 2.2.4. ***rac-(4-((2,2-Dimethyl-1,3-dioxolan-4-yl)methoxy)phenyl)methanethiol (rac-S4)***

To a solution of compound ***rac*-S3** (332 mg, 1.12 mmol) in anhydrous methanol (15 mL) was added dry K_2_CO_3_ (185 mg, 1.34 mmol) and the resulting suspension was stirred for 15 min at room temperature under an argon atmosphere. The reaction mixture was then neutralised by dropwise addition of 2M HCl aqueous solution and the solvent was removed under reduced pressure. The resulting residue was dissolved in distilled water (15 mL) and extracted with dichloromethane (2 × 10 mL). The combined organic layers were dried with anhydrous MgSO_4_, filtered and evaporated under reduced pressure. The resulting residue was purified by column chromatography (eluent: hexane/Et_2_O 4:1) to afford compound ***rac*-S4** (241 mg, 85% yield) as a white oily solid. **mp**: 33–34 °C. **IR** (KBr, ν_max_/cm^−1^): 1456, 1513, 1611. **^1^H-NMR** (400 MHz, CDCl_3_, δ): 7.26–7.20 (m, 2H), 6.89–6.82 (m, 2H), 4.51–4.43 (m, 1H), 4.16 (dd, 1H, J = 8.4, J = 6.4), 4.04 (dd, 1H, J = 9.6, J = 5.6), 3.92 (dd, 1H, J = 9.6, J = 6.0), 3.89 (dd, 1H, J = 8.4, J = 5.8), 3.70 (d, 2H, J = 7.6), 1.73 (t, 1H, J = 7.6), 1.46 (s, 3H), 1.40 (s, 3H). **^13^C-NMR** (100 MHz, CDCl_3_, δ): 157.6, 133.7, 129.1, 114.7, 109.7, 73.9, 68.9, 66.8, 28.3, 26.8, 25.3. **HRMS (ESI^+^)**: *m*/*z* [M + Na]^+^ calculated for C_13_H_18_NaO_3_S 277.0870, found 277.0870.

#### 2.2.5. ***rac-4-((4-((2,2-Dimethyl-1,3-dioxolan-4-yl)methoxy)benzyl)thio)-7-nitrobenzo[c][1,2,5]oxadiazole (rac-S5)***

To a solution of 4-chloro-7nitrobenzofurazan (285 mg, 1.43 mmol) and compound ***rac*-S4** (242 mg, 0.95 mmol) in anhydrous DMF (22 mL) was added anhydrous pyridine (0.15 mL, 1.90 mmol) and the reaction mixture was stirred for 2 h at 80 °C under an argon atmosphere. An extra amount of anhydrous pyridine (77 µL, 0.95 mmol) was then added and the mixture was stirred for 2 additional hours under the same reaction conditions. The solvent was removed under reduced pressure and the resulting residue was purified by column chromatography (eluent 1: hexane/Et_2_O 7:3, eluent 2: dichloromethane/hexane 9:1; eluent 3: dichloromethane/Et_2_O 9:1) to afford compound ***rac*-S5** (178 mg, 45% yield) as a yellow solid. **mp**: 106–107 °C. **IR** (KBr, ν_max_/cm^−1^): 1304, 1330, 1512. **^1^H-NMR** (400 MHz, CDCl_3_, δ): 8.34 (d, 1H, J = 8.0), 7.38–7.31 (m, 2H), 7.18 (d, 1H, J = 8.0), 6.93–6.86 (m, 2H), 4.51–4.43 (m, 1H), 4.47 (s, 2H), 4.16 (dd, 1H, J = 8.4, J = 6.4), 4.04 (dd, 1H, J = 9.6, J = 5.6), 3.93 (dd, 1H, J = 9.6, J = 5.6), 3.89 (dd, 1H, J = 8.4, J = 5.6), 1.45 (s, 3H), 1.39 (s, 3H). **^13^C-NMR** (100 MHz, CDCl_3_, δ): 158.6, 149.1, 142.4, 141.0, 132.8, 130.6, 130.0, 125.9, 121.3, 115.1, 109.8, 73.9, 68.8, 66.7, 36.2, 26.8, 25.3. **HRMS (ESI^+^)**: *m*/*z* [M + Na]^+^ calculated for C_19_H_19_NaN_3_O_6_S 440.0887, found 440.0879.

#### 2.2.6. ***rac-3-(4-(((7-Nitrobenzo[c][1,2,5]oxadiazol-4-yl)thio)methyl)phenoxy)propane-1,2-diol (rac-*IV-j*)***

Bismuth trichloride (26 mg, 0.082 mmol) and 7 drops of distilled water were sequentially added to a solution of compound ***rac*-S5** (171 mg, 0.41 mmol) in acetonitrile (8.0 mL). The reaction mixture was stirred for 5 h at room temperature. The solvent was then removed under reduced pressure. The resulting residue was dissolved in MeOH/AcOEt 1:1 and adsorbed in silica-gel to be purified by column chromatography (eluent 1: Et_2_O, eluent 2: AcOEt), affording compound ***rac*-IV-j** (136 mg, 88% yield) as a yellow solid. **mp**: 151–152 °C. **IR** (KBr, ν_max_/cm^−1^): 1304, 1338, 1510, 3289, 3389. **^1^H-NMR** (400 MHz, acetone-d_6_, δ): 8.56 (d, 1H, J = 8.0), 7.63 (d, 1H, J = 8.0), 7.52–7.46 (m, 2H), 7.00–6.93 (m, 2H), 4.66 (s, 2H), 4.14–4.05 (m, 2H), 4.02–3.93 (m, 2H), 3.83–3.77 (m, 1H), 3.71–3.60 (m, 2H). **^13^C-NMR** (100 MHz, acetone-d_6_, δ): 159.9, 150.2, 143.8, 141.1, 132.5, 131.3, 127.3, 123.1, 115.8, 71.3, 70.4, 64.1, 36.1. **HRMS (ESI^+^)**: *m*/*z* [M + Na]^+^ calculated for C_16_H_15_NaN_3_O_6_S 400.0574, found 400.0577.

#### 2.2.7. ***rac-3-(4-(((7-Aminobenzo[c][1,2,5]oxadiazol-4-yl)thio)methyl)phenoxy)propane-1,2-diol (rac-*IV-k*)***

To a suspension of compound ***rac*-S5** (71 mg, 0.17 mmol) in EtOH/AcOH/H_2_O 2:2:1 (3.5 mL) was added iron powder (49 mg, 0.88 mmol) and the reaction mixture was sonicated (80 W, 45 kHz) at 30 °C for 30 min. The reaction mixture was then filtered through a pad of celite^®^ and the filtrate was diluted with dichloromethane (10 mL) and washed with saturated K_2_CO_3_ aqueous solution (2 × 10 mL). The combined aqueous layers were extracted with dichloromethane (10 mL). The combined organic layers were dried with anhydrous MgSO_4_, filtered and evaporated under reduced pressure. The resulting crude was used in the next reaction step without further purification. Bismuth trichloride (11 mg, 0.034 mmol) and 3 drops of distilled water were sequentially added to a solution of the former crude compound in acetonitrile (3.5 mL). The reaction mixture was stirred for 16 h at room temperature. The solvent was then removed under reduced pressure. The resulting residue was dissolved in MeOH/AcOEt 1:1 and adsorbed in silica-gel to be purified by column chromatography (eluent 1: Et_2_O, eluent 2: Et_2_O/MeOH 95:5), affording compound ***rac*-IV-k** (44 mg, 75% yield) as an orange solid. **mp**: 161–162 °C. **IR** (KBr, ν_max_/cm^−1^): 3248, 3358, 3453. **^1^H-NMR** (400 MHz, CD_3_OD, δ): 7.08 (d, 1H, J = 7.6), 7.05–6.98 (m, 2H), 6.81–6.73 (m, 2H), 6.19 (d, 1H, J = 7.6), 4.07 (s, 2H), 4.01–3.96 (m, 1H), 3.96–3.86 (m, 2H), 3.66 (dd, 1H, J = 11.2, J = 4.8), 3.61 (dd, 1H, J = 11.2, J = 5.2). **^13^C-NMR** (100 MHz, CD_3_OD, δ): 159.4, 152.4, 146.5, 141.3, 138.6, 131.6, 131.1, 115.3, 107.0, 106.3, 71.8, 70.3, 64.1, 39.2. **HRMS (ESI^+^)**: *m*/*z* [M + Na]^+^ calculated for C_16_H_17_NaN_3_O_4_S 370.0832, found 370.0853.

#### 2.2.8. ***rac-3-(4-(((7-Aminobenzo[c][1,2,5]oxadiazol-4-yl)sulfinyl)methyl)phenoxy)propane-1,2-diol (rac-*IV-l*)***

To a solution of compound ***rac*-IV-k** (24 mg, 0.07 mmol) in glacial acetic acid (2.0 mL) was added 35% H_2_O_2_ aqueous solution (57 µL, 0.59 mmol) and the reaction mixture was stirred for 1.5 h at room temperature. The reaction mixture was quenched with 1M Na_2_SO_3_ aqueous solution (0.59 mL, 0.59 mmol) and the resulting suspension was diluted with distilled water and extracted with ethyl acetate (4 × 10 mL). The combined organic layers were dried with anhydrous MgSO_4_, filtered and evaporated under reduced pressure. The resulting residue was dissolved in methanol and adsorbed in silica-gel to be purified by column chromatography (eluent 1: AcOEt, eluent 2: Et_2_O/MeOH 9:1), affording compound ***rac*-IV-l** (21 mg, 82% yield) as a yellow solid. **mp**: 73–74 °C. **IR** (KBr, ν_max_/cm^−1^): 1032, 3405. **^1^H-NMR** (400 MHz, CD_3_OD, δ): 7.39 (d, 1H, J = 7.6), 6.99–6.92 (m, 2H), 6.85–6.78 (m, 2H), 6.31 (d, 1H, J = 7.6), 4.54 (d, 1H, J = 13.0), 4.39 (d, 1H, J = 13.0), 4.03–3.96 (m, 1H), 3.96–3.87 (m, 2H), 3.70–3.58 (m, 2H). **^13^C-NMR** (100 MHz, CD_3_OD, δ): 160.6, 147.3, 146.1, 142.9, 138.1, 132.6, 122.9, 115.6, 111.8, 104.1, 71.7, 70.3, 64.1, 59.4. **HRMS (ESI^+^)**: *m*/*z* [M + Na]^+^ calculated for C_16_H_17_NaN_3_O_5_S 386.0781, found 386.0779.

### 2.3. Bacterial Strains, Culture Media and Growth Conditions

*H. pylori* (ATCC 700392) and *Helicobacter hepaticus* (ATCC 51449) were purchased from the American Type Culture Collection (ATCC, Manassas, VA, USA), whereas *Campylobacter jejuni* (ATCC 33560) was donated by Dr. Pilar Mañas from the University of Zaragoza (Spain). Strains of *Helicobacter felis* (JKM5), *Helicobacter suis* (HS1 and HS5), *Helicobacter heilmannii* (ASB1.4 and ASB2), *Helicobacter ailurogastricus* (ASB7 and ASB9) and *Helicobacter bizzozeronii* (ASB22 kol15) have been described in Smet et al. [21]. Strains of *H. bizzozeronii* (10 and Heydar) were provided by Prof. Dr. Mirko Rossi. *Helicobacter muridarum* and *Helicobacter bilis* were available from Dr. Eliette Touati from the Institut Pasteur (Paris, France). *Salmonella enterica* subsp. *enterica* serovar Typhimurium (*S.* Typhimurium; SV 5015), *Escherichia coli* (ATCC 10536)*, Pseudomonas aeruginosa* (ATCC 15442)*, Bacillus* sp. (CECT 40)*, Streptococcus pneumoniae* (ATCC 49619)*, Listeria monocytogenes* (ATCC BAA-679)*, Enterococcus faecalis* (JH2-2)*, Corynebacterium diphtheriae* (ATCC 39255)*, Corynebacterium ammoniagenes* (ATCC 6872), *Mycolicibacterium smegmatis* (ATCC 700084) and *Staphylococcus aureus* (ATCC 29213) were available from the culture collections of the Departments of Microbiology and Biochemistry of the University of Zaragoza (Spain).

The strains *P. aeruginosa* MPAO1 and *P. aeruginosa* PW9682, a Δ*rmlC* mutant of MPAO1 that is defective in lipopolysaccharide (LPS) biosynthesis [22], were obtained from the Transposon Mutant Collection of the University of Washington [23,24]. *Klebsiella pneumoniae* subspecies *pneumoniae* strains 3025 (serotype O1:K^−^, Str^R^; produces smooth-type LPS with d-galactan I and d-galactan II O-antigens) and CWK43 (*rpsL*, *cps*, *rfb*, Str^R^, serotype O^−^:K^−^; produces truncated rough-type LPS) [25,26,27] were kindly provided by Prof. Dr. Ian R. Poxton (Department of Medical Microbiology, University of Edinburgh, Edinburgh, U.K.) and Prof. Dr. Chris Whitfield (Department of Molecular and Cellular Biology, University of Guelph, Guelph, ON, Canada), respectively. The *Stenotrophomonas maltophilia* K279a [28] wild-type strain was originally obtained from the laboratory of Prof. Dr. Matthew B. Avison (School of Medical Sciences, University of Bristol, Bristol, U.K.), and the construction of the *S. maltophilia* K279a Δ*rmlBACD* [29] mutant lacking the O-antigen of LPS has been described elsewhere.

Cultures of *H. pylori, H. hepaticus*, *H. muridarum* and *H. bilis* were grown in blood agar base No. 2 (Oxoid, Basingstoke, Hampshire, UK) supplemented with 8% defibrinated horse blood (Oxoid) under microaerophilic conditions (85% N_2_, 10% CO_2_, 5% O_2_) at 37 °C for 48–72 h. For drug susceptibility testing, bacteria were grown under the same conditions in brain heart infusion (BHI) broth (Oxoid) supplemented with 4% foetal bovine serum (FBS) (Pan-Biotech, Aidenbach, Germany) for *H. pylori,* 10% FBS and 2.5 g/L yeast extract (Scharlab, Barcelona, Spain) for *H. hepaticus*, and 10% FBS for *H. muridarum* and *H. bilis.* In these last two cases, the cultures were also stirred at 150 rpm. Cultures of *C. jejuni* were grown under the same conditions except for the FBS supplement and shaking. Susceptibility of *H. felis* and *H. bizzozeronii* to the compounds was evaluated by incubating bacteria on Mueller-Hinton II agar (Becton Dickinson, Cockeysville, MD, USA), supplemented with 10% defibrinated horse blood and 0.6% Vitox (Oxoid) for 7 days at 37 °C under microaerophilic conditions. *H. suis*, *H. heilmannii* and *H. ailurogastricus* were grown for 48 h at pH 5 and 37 °C under microaerophilic conditions, using a biphasic medium consisting of Brucella agar (BD, Franklin Lakes, NJ, USA) supplemented with 20% inactivated fetal calf serum (Hyclone, ThermoFisher Scientific, Waltham, MA, USA), Vitox supplement (Oxoid), and *Campylobacter* selective supplement (Skirrow, Oxoid), with Brucella broth (Oxoid) added on top. On the other hand, *S.* Typhimurium, *E. coli, P. aeruginosa, Bacillus* sp. and *E. faecalis* were grown under aerobic conditions at 37 °C in lysogeny broth (LB) overnight with stirring at 150 rpm. Same conditions were used for *M. smegmatis* cultures, but it was incubated for 72 h without shaking. Cultures of *L. monocytogenes, C. diphtheriae* and *C. ammoniagenes* were grown in BHI broth for 24 h with stirring at 150 rpm, with the exception of the last one whose culture was incubated overnight. *S. pneumoniae* was grown in BHI broth supplemented with 4% FBS for 10 h, while *S. aureus* was cultured in Mueller-Hinton broth (Panreac, Castellar del Vallès, Spain) pH 7 for 16 h. Finally, the strains 3025 and CWK43 of *K. pneumoniae*, K279a and K279a Δ*rmlBACD* of *S. maltophilia*, and MPAO1 of *P. aeruginosa* were routinely grown with shaking (220 rpm) at 37 °C in LB medium. For cultivation of *P. aeruginosa* PW9682, the LB medium was supplemented 17 µg/mL of tetracycline.

### 2.4. Evaluation of the Antibacterial Activity against a Diverse Microbial Panel

Serial broth microdilutions were made to determine the Minimal Inhibitory Concentration (MIC) of **IV**, **IV-a**, **IV-b**, **IV-c**, **IV-d**, ***rac*-IV-j**, ***rac*-IV-k**, ***rac*-IV-l**, **Mnz** and **Cla** against *H. pylori*, *H. hepaticus*, *H. muridarum, H. bilis, C. jejuni*, *S.* Typhimurium, *E. coli, P. aeruginosa, Bacillus* sp., *S. pneumoniae*, *L. monocytogenes*, *E. faecalis*, *S. aureus*, *C. diphtheriae*, *C. ammoniagenes* and *M. smegmatis* as previously described [18]. Briefly, each well of a 96-well plate was inoculated with 100 μL of fresh liquid bacterial culture at 10^6^ CFU/mL (for *Helicobacter* strains) or 5 × 10^5^ CFU/mL (for the other bacteria), except for the first well of each raw, which received 200 μL of bacterial culture plus 2 μL of compound (from stock solutions at 6.4 mg/mL in 100% DMSO). After performing two-fold serial dilutions, plates were incubated under the culture conditions described above, to evaluate the antimicrobial activity of compounds in a concentration range of 0.031–64 μg/mL. MIC values, defined as the lowest concentration of compound able to inhibit bacterial growth, were determined after addition of 30 μL of resazurin 0.1 mg/mL (Sigma-Aldrich, San Luis, MO, USA). Positive and negative controls were included in all the experiments, which were performed, at least, twice in duplicate. MIC values of omeprazole, rabeprazole, CCCP, reserpine and valinomycin against *H. pylori* (ATCC 700392) were also determined by the microdilution MIC testing as explained above.

Susceptibility of *H. felis* and *H. bizzozeronii* to compounds **IV** and ***rac*-IV-j** was evaluated by the agar dilution method, as previously described [30]. Succinctly, compounds were added to agar plates according to two-fold serial dilutions, with final concentrations ranging from 0.03 to 128 μg/mL. Agar plates free of compounds were included as positive controls. *H. felis* and *H. bizzozeronii* bacteria were grown for 72 h, then harvested and suspended in sterile saline to a density of 3 on the McFarland turbidity scale. Then, plates were seeded by a Steers inoculum replicator (MAST, London, United Kingdom) and incubated in a microaerophilic atmosphere. After 7 days, plates were read, the MIC being determined as the lowest compound concentration that inhibits visible growth. MICs of **IV** and ***rac*-IV-j** against *H. suis*, *H. heilmannii* and *H. ailurogastricus* were determined by using a combined agar and broth dilution method in 24-well plates (Greiner Bio-On, Frickenhausen, Germany), as previously described [31]. In brief, agar plates and broth were prepared to contain two-fold serial dilutions of the tested compounds. Then, they were inoculated with 150 µL of the bacterial culture at 5 × 10^7^ bacteria/mL, so that each well of the 24-well plates contained 200 μL of broth and 400 μL agar, with final concentrations of the compounds ranging from 0.03 to 128 μg/mL. Wells containing only bacterial culture were included as positive controls. MICs were determined as the lowest compound concentration with at least 50% bacterial growth inhibition compared to controls. To determine the impact of culture and pH conditions on the activity of the compounds **IV** and ***rac*-IV-j**, 2 different MIC assays were performed for *S. aureus*: (i) the combined agar and broth dilution method at pH 5, similar to the described above and (ii) the broth microdilution method (according to the Clinical and Laboratory Standards Institute ([32]) standards) in unsupplemented Mueller Hinton broth at pH 7, as previously reported [31]. In both cases, bacterial growth was analysed after 16–24 h of incubation. The MIC was recorded as the lowest compound concentration for which there was no turbidity. Susceptibility of *P. aeruginosa* strains MPAO1 and PW9682, *S. maltophilia* strains K279a and K279a Δ*rmlBACD*, as well as *K. pneumoniae* 3025 and CWK43 to **IV**, **IV-a**, **IV-b**, **IV-c** and **IV-d** was studied by MIC determination using the broth microdilution method in LB medium essentially as described above, except that each culture with a starting OD_600_ of 0.01 was mixed with a two-fold serial dilution of each compound in a concentration range between 0.25 and 512 μg/mL. The microtiter plates were incubated at 37 °C in a humidified chamber for 20 h, followed by determination of the OD_600_ values and incubation of the cultures in the presence of resazurin at 37 °C for 30 min.

### 2.5. Cloning, Expression, Purification and Quantification of Recombinant Flavodoxin

Recombinant wild-type *Hp*-Fld, along with N14A, A55W, V113W, Q115W, T116W, K133A and D142Y mutants, were overexpressed in *E. coli* strain BL21 (DE3, Sigma-Aldrich) and purified as previously described [11] with slight modifications. A bacterial culture containing the pET28a-*fldA* plasmid was grown at 37 °C in LB medium supplemented with 20 μg/mL kanamycin (Sigma-Aldrich) to an OD_600_ of 0.8. Then, flavodoxin expression was induced by addition of 1 mM isopropyl β-d-thiogalactoside (Thermo Fisher Scientific) and further incubation at 37 °C overnight. After centrifugation, bacteria were resuspended in cell disruption buffer (50 mM Tris-HCl buffer, 100 μM EDTA, 100 μM β-mercaptoethanol and 1 μM phenylmethanesulphonyl fluoride, pH 8), mixed with 50 mg of FMN (Santa Cruz Biotechnology, Dallas, TX, USA) and lysed by sonication (Hielscher UP200S, 24 kHz). After centrifugation at 18.000 rpm (in an Avanti J-26XP High Performance Centrifuge (Beckman Coulter, Brea, CA, USA) with a JA-25.50 rotor) for 30 min at 4 °C, the supernatant was precipitated with 65% (NH_4_)_2_SO_4_ and centrifuged at 18.000 rpm for 30 min at 4 °C. The supernatant was then loaded onto a diethylaminoethyl (DEAE) cellulose column equilibrated with 65% (NH_4_)_2_SO_4_ in 50 mM Tris–HCl buffer, pH 8, and the flavodoxin, which is only weakly bound to the column, was eluted with 65% (NH_4_)_2_SO_4_ in 50 mM Tris–HCl buffer, pH 8 and then dialysed. The eluted fractions were poured onto a DEAE column equilibrated with 50 mM Tris–HCl buffer, pH 8 and the protein was eluted with a linear gradient from 0 to 1 M NaCl in 50 mM Tris–HCl buffer, pH 8. Then, it was dialysed against the corresponding buffer, concentrated, transformed into holoprotein if needed, and quantified by using the theoretical extinction coefficients (ε_APO, 278 nm_ = 15.96 mM^−1^cm^−1^; ε_HOLO, 278 nm_ = 37.37 mM^−1^cm^−1^; ε_HOLO, 452 nm_ = 10.65 mM^−1^cm^−1^). Flavodoxin purity was determined by 15% SDS-PAGE, followed by Coomassie staining (Figure S1). The native conformation of wild type and of the flavodoxin variants can be inferred from the corresponding visible spectra (Figure S2) as, in all variants, the distinctive flavin absorption shoulder at 480 nm, characteristic of FMN bound to native flavodoxin, is clearly observed [33]. 

### 2.6. Determination of the Hp-Fld-Binding Affinity of Inhibitors

Isothermal titration calorimetry (ITC) experiments were carried out in an Auto-iTC200 calorimeter (MicroCal, Malvern-Panalytical, Malvern, UK) in order to characterise the affinity of the wild-type *Hp*-Fld protein and the different mutants N14A, A55W, V113W, Q115W, T116W, K133A and D142Y for the different compounds. Recombinant *Hp*-Fld at 10–20 µM concentration in 50 mM EPPS buffer, pH 9 (for **IV**, **IV-a**, **IV-b**, **IV-c**, ***rac*-IV-j**, ***rac*-IV-k** and ***rac*-IV-l**), or in 50 mM Tris-HCl buffer, pH 9 (for **IV-d**) was titrated with 120–300 µM ligand solutions in the same buffer, prepared from stock compound solutions in 100% DMSO. At the residual DMSO concentration of 2.5% employed in the working solutions, wild-type flavodoxin has been described to be conformationally stable and biochemically active [16]. Thermodynamic parameters of the binding equilibrium were calculated by non-linear least-squares regression analysis considering a single ligand binding site and using the user-defined fitting routines in the MicroCal LLC ITC module from the Origin 7.0 software (Northampton, MA, USA).

### 2.7. Molecular Docking of Compounds ***IV***, ***IV-a***, ***IV-b***, ***IV-c*** and ***IV-d*** to Hp-Fld

Initial structures of compounds **IV**, **IV-a**, **IV-b**, **IV-c** and **IV-d** were obtained from their SMILES codes through the online NCI/CADD SMILES translator tool (https://cactus.nci.nih.gov/translate/ (accessed on 10 September 2020), NIH National Cancer Institute, Frederick, MD, USA). They were subsequently optimised at the ab initio level (#HF/6-31G*) with Gaussian 09 [34], and the resulting structures were docked onto the three-dimensional structure of wild-type *Hp*-Fld (PDB ID: 1FUE) with AutoDock4.2.6 (La Jolla, CA, USA) [35]. A first round of docking was performed, allowing the compounds to sample the whole protein surface (blind docking), keeping the protein rigid and allowing the compounds’ rotatable bonds to freely rotate. The flavodoxin regions, which showed the highest scored poses of docked compounds, were then subjected to targeted docking (local sampling), now with both entities in flexible mode (protein sidechains’ and compounds’ rotatable bonds allowed to rotate). The amino acid residues that appeared crucial for the binding were identified. They were replaced by site directed mutagenesis for the sake of testing their potential contribution to complex formation with the inhibitory compounds by ITC. To that end, the affinity of the complexes formed by the chosen compounds and wild-type *Hp*-Fld was compared to that of the complexes established with the mutant flavodoxins.

### 2.8. Combined Antimicrobial Effect

Once the MICs of EIs CCCP, reserpine and valinomycin against *H. pylori* were determined, as described above, the antimicrobial effect of several **IV**-related compounds was evaluated in combination with these EIs. To that end, the MIC of each compound against a *H. pylori* culture was evaluated in the presence of each EI at a concentration of ¼ of its MIC. The assay was carried out in the same way as a conventional microdilution MIC testing, and the culture conditions were as described above. We considered that any EI had a significant effect on a compound MIC when this was reduced at least four-fold in the presence of that EI. Each experiment was performed twice in triplicate.

### 2.9. Generation of Spontaneous Resistant Mutants

*H. pylori* reference strain ATCC 700392, at 5 × 10^6^ and 5 × 10^7^ CFU/mL in BHI broth (Oxoid) supplemented with 4% FBS, was incubated at 37 °C for 5 days under microaerophilic conditions in the presence of compounds **IV**, **IV-d** and ***rac*-IV-l** at final concentrations of 32, 16, 8, 4, 2, 1 and 0.5 times their MICs. Because *H. pylori* is able to develop resistance to **Mnz**, this antibiotic was used as a control. The generation of resistant mutants was evaluated by the MIC values of compounds **IV**, **IV-a**, **IV-c**, **IV-d, *rac*-IV-l** and **Mnz** against the treated cultures, compared to the corresponding MIC of the parental wild-type strain. The experiment was performed twice in duplicate.

### 2.10. Chequerboard Synergy Testing

Pairwise interactions between several IV-related compounds and **Mnz**, **Cla**, omeprazole or rabeprazole, as well as between parent compound **IV** and its derivatives **IV-a**, **IV-b**, **IV-c**, **IV-d**, ***rac*-IV-j**, ***rac*-IV-k** and ***rac*-IV-l** were studied in *H. pylori* strain ATCC 700392. In a 96-well plate containing a fresh culture of bacteria at 10^6^ CFU/mL, one compound (at 32 times its MIC) was added to the second column and the other one (at 4 times its MIC) was poured in raw G. Both compounds were serially 2-fold diluted to generate a gradient matrix of both compounds. The first and last columns of the plate allowed one to test each compound alone. After incubation as described above for *H. pylori* growth, plates were treated with resazurin, as in a conventional MIC assay, to reveal the result of the interaction effect. For each pair of compounds, the fractional inhibitory concentration index (FICI) was determined as FICI = FIC_1_ + FIC_2_, where FIC_1_ = (MIC_compound 1_ in presence of compound 2)/(MIC_compound 1_ alone) and FIC_2_ = (MIC_compound 2_ in presence of compound 1)/(MIC_compound 2_ alone). The value of the FICI indicates whether two compounds show a synergistic effect (FICI ≤ 0.5), no interaction (0.5 < FICI ≤ 4) or an antagonistic effect (FICI > 4) effect [36]. Pairs of FIC values can be represented graphically, and a concave curve indicating synergy. The point of the curve closest to the intersection of the axes corresponds to the most effective combination of compounds that inhibits *H. pylori* growth [37,38]. 

## 3. Results

### 3.1. Selectivity of the Flavodoxin Inhibitors for Bacteria of the Helicobacter Genus

The in vitro efficacy of **IV**-related compounds, **Mnz** and **Cla,** was evaluated (Table 1) against several bacteria from different phyla (Table S2). Their therapeutic activity was reported as the lowest concentration, leading to 50% bacterial growth inhibition in comparison with drug-free controls (for the *Helicobacter* species requiring a biphasic growth medium) or as the MIC (for the other bacteria tested in broth). As seen in Table 1, while *H. pylori* is susceptible to all IV-related compounds (except ***rac*-IV-l**) at low concentrations, the growth of many other bacteria tested is not inhibited by most of these derivatives even at concentrations up to 64 or 512 μg/mL, respectively. However, according to MIC breakpoints of antibiotics traditionally used to treat these bacterial infections [39], two compounds, **IV** and ***rac*-IV-j**, show moderate to good antimicrobial activity (≤32 µg/mL) against *C. jejuni*, and the Gram-positive *Bacillus* sp., *S. pneumoniae*, *E. faecalis*, *S. aureus*, *C. diphtheriae* and *C. ammoniagenes*. Moreover, these compounds show low MICs for the gastric non-*H. pylori Helicobacter* species tested (*H. felis*, *H. suis*, *H. heilmannii*, *H. ailurogastricus* and *H. bizzozeronii)* and against the enterohepatic *Helicobacter* species *H. muridarum* and *H. bilis.*

Globally, the effect of the compounds can be described considering three groups of bacteria: Gram-positive, the Gram-negative bacteria belonging to the *Helicobacter* and *Campylobacter* genera, and all other Gram-negative ones. The growth of most Gram-positive bacteria tested —except *M. smegmatis* and *L. monocytogenes*— is inhibited by compounds **IV** and ***rac*-IV-j** but not by compounds **IV-a**, **IV-b**, **IV-c**, **IV-d**, ***rac*-IV-k** or ***rac*-IV-l**.

The other Gram-negative bacteria tested, which belong to the class gamma-proteobacteria, are generally non-susceptible to any of the compounds, including isogenic LPS mutants of *P. aeruginosa*, *S. maltophilia* and *K. pneumoniae* tested to address potential permeabilization issues of the compounds across the outer membrane of the parental wild-type strains. However, most Gram-negative bacteria of the *Helicobacter* genus (9 species tested) and the closely related *C. jejuni* species (they both belong to the class epsilon-proteobacteria and the order *Campylobacterales*) are inhibited, at least by compounds **IV** and ***rac*-IV-j**. Within this group, *H. pylori* is inhibited by all the compounds tested (lead compound **IV** was initially discovered as an *Hp*-Fld inhibitor); the five remaining gastric species (*H. felis, H. suis, H. heilmannii, H. ailurogastricus* and *H. bizzozeronii*) are inhibited at least by compounds **IV** and ***rac*-IV-j** at concentrations ≤32 µg/mL (the other compounds have not been tested in these species); and two out of three enterohepatic species tested (*H. hepaticus, H. muridarum* and *H. bilis)* are inhibited by compounds **IV** and ***rac*-IV-j,** but not by the rest of the compounds.

### 3.2. Probable Interaction of the Inhibitors at a Pocket near the FMN Binding Site

Interaction between wild-type *Hp*-Fld and compounds **IV**, **IV-a**, **IV-b**, **IV-c**, **IV-d**, ***rac*-IV-j**, ***rac*-IV-k** and ***rac*-IV-l** was evaluated by ITC (Figure S3 and Table S1). These studies reveal only small differences in the binding affinities of the compounds to the protein target, as they all exhibit dissociation constants in the micromolar range and a 1:1 protein:ligand stoichiometry. As shown, the interaction between wild-type *Hp*-Fld and **IV**-derivatives is entropically driven, and characterised by a moderate affinity. The small contribution of the enthalpy balance is, in some cases, positive and in some others negative, which gives rise to the different tendencies of the binding curves.

**Figure 3:**
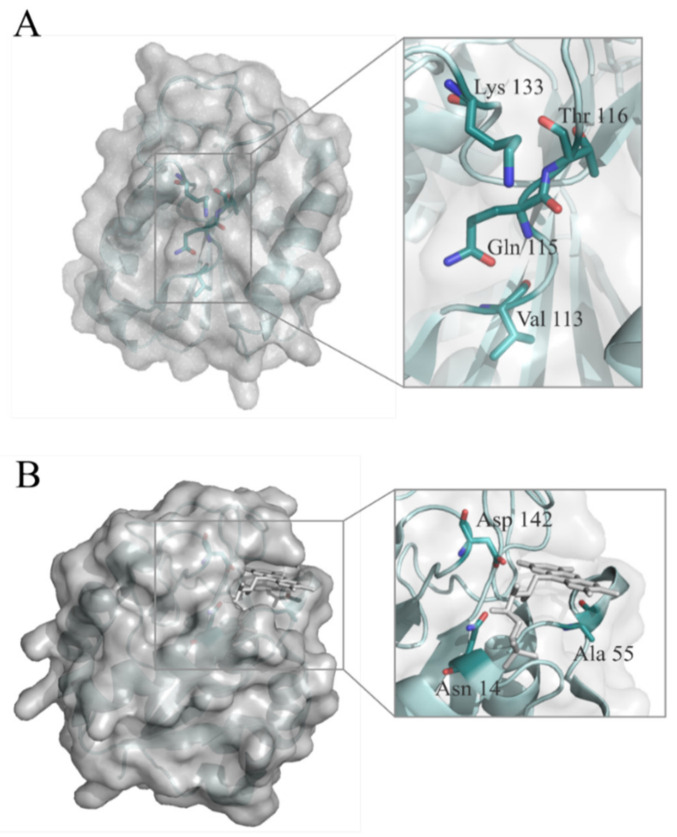
Molecular surface and ribbon models showing putative interacting residues of wild-type *Hp*-Fld with compounds **IV**, **IV-a**, **IV-b**, **IV-c** and **IV-d**. (**A**): Val 113, Gln 115, Thr 116 and Lys 133, predicted by molecular docking, and (**B**): Asn 14, Ala 55 and Asp 142, close to the structural pocket near the cofactor binding site. The FMN cofactor is represented as grey sticks and the nitrogen and oxygen atoms from the amino acid residues are depicted as red and blue sticks, respectively.

Despite considerable efforts, the crystallization of a complex between *Hp*-Fld and any of the inhibitors has not been possible yet. Considering the lack of structural information on flavodoxin-inhibitor complexes, molecular docking assays were carried out to try to understand the structural basis of the interaction between the protein and the inhibitors. These studies proposed one main binding hotspot for the inhibitors, which was located at the long-loop characteristic of long-chain flavodoxins. In particular, the best binding poses for compounds **IV**, **IV-a**, **IV-b**, **IV-c** and **IV-d** showed interaction with residues V113, Q115, T116 and K133 of the protein (Figure 3A). On the other hand, *Hp*-Fld shows a characteristic pocket near the FMN binding site, where the binding of small compounds has been proposed, that could interfere either with electron transfer or with the interaction of flavodoxin with other protein partners [11]. The pocket arises from the unusual presence in *Hp*-Fld of an alanine residue (A55) at the si face of the cofactor, where other flavodoxins commonly bear a bulky residue. Three residues, namely: N14 and D142 (close to each other and interacting with the FMN phosphate and its ribityl moiety) and especially A55 (right at the pocket edge) have been noticed that could affect the binding of inhibitors at the pocket (Figure 3B).

**Figure 4:**
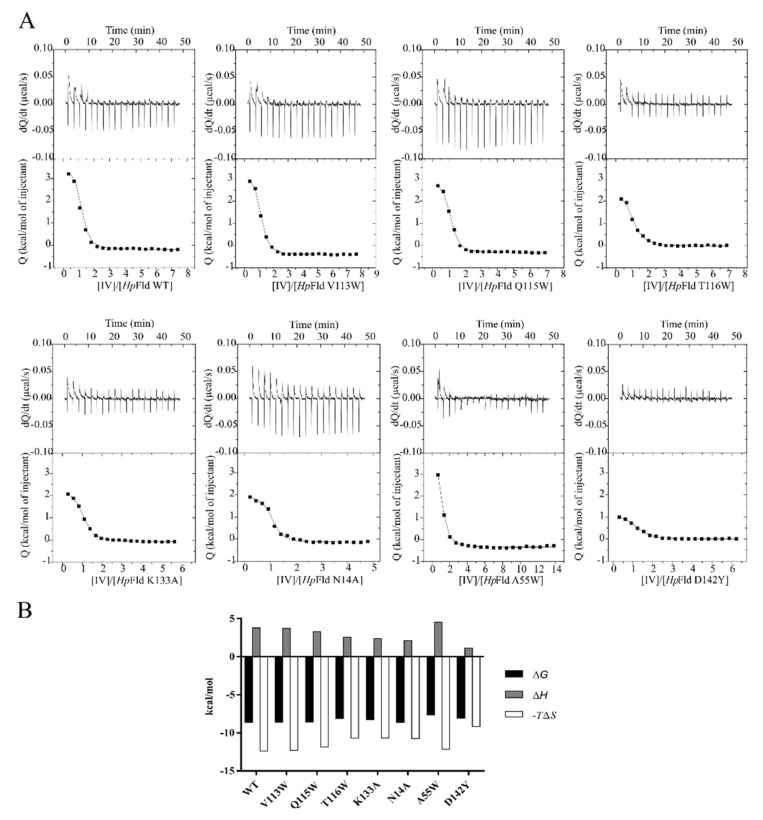
Thermodynamic analysis of the interaction between compound **IV** and wild-type *Hp*-Fld and mutants N14A, V113W, Q115W, T116W and K133A by ITC. (**A**) The upper plots show the thermograms (thermal power as a function of time), whereas the lower panels exhibit the binding isotherms (titrant normalised heat effects as a function of the ligand:protein molar ratio in the cell). In these last ones, the solid line corresponds to the best fit. (**B**) Thermodynamic parameters of each flavodoxin interaction with compound IV. Gibbs energy (Δ*G*), enthalpy (Δ*H*) and entropic contribution (−*T*Δ*S*) are represented in black, grey and white bars, respectively.

In order to assess whether the protein region predicted by the docking analysis is the likely binding site for the inhibitors, residues V113, Q115 and T116 were mutated to tryptophan, which may introduce steric hindrance, and K133 was mutated to alanine, which removes the positive charge of the lysine side chain. At the alternative potential binding site located near the cofactor, N14 was replaced by an alanine in order to remove its hydrogen bonding groups, and A55 and D142 were replaced by bulkier tryptophan and tyrosine residues, respectively. The seven wild type residues replaced are located at the protein surface and the seven mutants *Hp*-Fld generated were expressed and purified with normal yields, and showed no indications of reduced conformational stability. The affinity of the complexes formed between wild-type or mutant *Hp*-Fld with compounds **IV** and **IV-c** was determined by ITC (Figure 4 and Figure S4).

The thermodynamic profiles of the interactions between wild-type and mutant *Hp*-Fld with either compound are similar. They indicate that all complexes are strongly stabilised by the binding entropy change and significantly destabilised by the enthalpy one (Table 2). The similarity of the thermodynamic profiles suggests that inhibitors **IV** and **IV-c** bind to all the mutant proteins at the same site as with the wild-type protein. Although no large changes in binding affinity occur in the mutants (Table 2), the data point to A55 as the amino acid residue whose replacement by a potentially interfering one cause the greatest changes in affinity. This is clearly seen in the complex between the protein and compound **IV**. In this complex, the replacement of A55 is the only one which decreases the affinity (as judged from the value of Δ*G* of binding) by more than 1 SD from the mean value exhibited by the eight *Hp*-Fld variants tested. Besides, the only substitution that makes the binding enthalpy of the complex even more unfavourable than in the wild-type protein is that of A55. The destabilization of the **IV-c** complex in the A55W mutant is not as large as in the **IV** complex, but its affinity is still below the mean of the **IV-c** complexes. Besides, if the affinity of each variant for **IV** and **IV-c** is averaged, the A55W mutant exhibits the weakest mean (−7.89 kcal/mol), being the only one below 1 SD of the mean of all inhibitors’ means (−8.24 ± 0.20 kcal/mol). Altogether, the ITC binding study does not confirm the putative binding site predicted by the docking studies, but supports, although non-conclusively, the hypothesis that the inhibitors bind at the pocket near the *Hp*-Fld cofactor.

**Figure 5:**
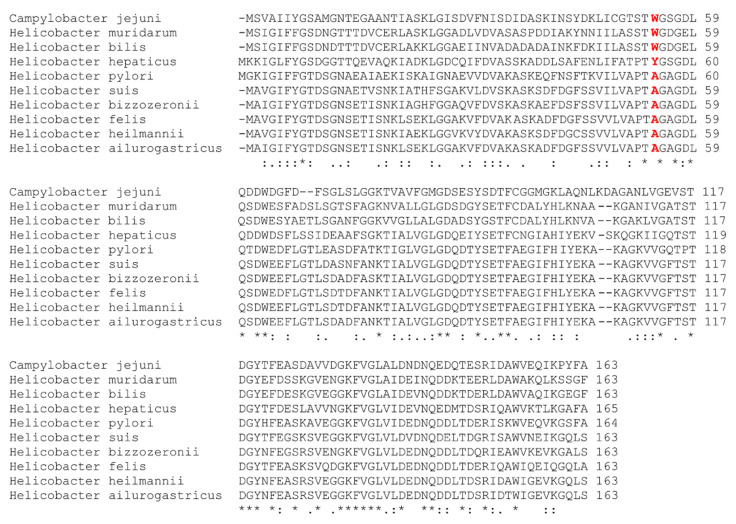
Multiple sequence alignment of flavodoxins from *C. jejuni*, *H. muridarum*, *H. bilis*, *H. hepaticus*, *H. pylori*, *H. suis*, *H. bizzozeronii*, *H. felis*, *H. heilmannii* and *H. ailurogastricus*. The alignment has been performed with Clustal Omega. [42] In all sequences, the amino acid residue at position 55 (according to *Hp*-Fld numeration) is highlighted in red. As seen, the flavodoxins from *H. pylori*, *H. suis*, *H. bizzozeronii*, *H. felis*, *H. heilmannii* and *H. ailurogastricus* exhibit an alanine at this position, whereas a tyrosine is shown in that from *H. hepaticus* and a tryptophan is exhibited by those from *C. jejuni*, *H. muridarum* and *H. bilis*. Asterisks (*) highlight positions with a fully conserved residue; colons (:) indicate positions of residues with high similarity; dots (.) indicate positions with residues possessing weak similarity.

These results are consistent with the fact that among the *Helicobacter* species (*H. pylori*, *H. hepaticus*, *H. muridarum* and *H. bilis*) plus *C. jejuni*, for which the whole battery of inhibitors has been tested (inhibitors **IV** to ***rac*-IV-l**), *H. pylori* is the only one bearing the alanine residue that generates the pocket near the cofactor (Figure 5), and the only one being inhibited by all the compounds tested (Table 1).

### 3.3. Prospects of a Low H. pylori Resistance Rate to ***IV***-Related Compounds

Bacterial efflux pumps are membrane proteins able to actively transport a number of substances, including drugs, from the cytoplasm to the extracellular environment. Efflux reduces the antibacterial activity of some drugs by throwing them out from the cell, and is considered as a low-level antibiotic resistance mechanism that can favour and promote the development of higher levels of resistance. In order to determine whether **IV**-related compounds are affected by efflux systems, the MICs of these molecules were evaluated in combination with sub-inhibitory concentrations of three different efflux inhibitors (EIs): CCCP, reserpine and valinomycin. To do that, we first determined the MICs of these EIs against *H. pylori* reference strain ATCC 700392. Those MICs turned out to be of 0.32, 12.5 and 10 μg/mL for CCCP, reserpine and valinomycin, respectively. Each EI was added at a concentration of ¼ MIC, and it was considered that a compound could be significantly transported out by efflux pumps when its MIC was decreased by at least 4 times. As shown in Table 3, CCCP, reserpine and valinomycin were able to reduce the MIC of **IV** by a factor of 4, indicating that compound **IV** could be affected by efflux, although this effect would be just in the limit of what we consider as significant. In addition, reserpine and valinomycin lowered the MIC of **IV-a** by half, an effect which is also achieved by ***rac*-IV-k** when it was combined with valinomycin, suggesting that efflux could play a weak role (but not significant enough) in the activity of these compounds. On the other hand, the same MICs, or even moderately higher (two- to four-fold) MICs, were obtained for compounds **IV-b**, **IV-c**, **IV-d** and ***rac*-IV-j**, which indicates that the EIs tested would interfere moderately with the antimicrobial activity of *Hp-Fld* inhibitors.

Altogether, these data showed that the antibacterial activity of **IV**-related compounds was not altered significantly by EIs, suggesting that the efflux pumps inhibited by those EIs were not able to actively transport any of the compounds of this family to a significant extent. Therefore, the intrinsic resistance of *H. pylori* to therapies based on these flavodoxin inhibitors can be regarded as moderate–low, which would make the development of higher resistance levels less likely.

In support of the latter observation, we were not able to select spontaneous mutants showing resistance to flavodoxin inhibitors. After incubating *H. pylori* strain ATCC 700,392 (at 5 × 10^6^ and 5 × 10^7^ CFU/mL) in the presence of different concentrations (0.5, 1, 2, 4, 8, 16 and 32 times their MICs) of compounds **IV**, **IV-d**, ***rac*-IV-l** and **Mnz** (as a control drug), the generation of spontaneous mutants was checked by MIC testing against those cultures. Whereas no significant changes in the MICs of compounds **IV**, **IV-d** and ***rac*-IV-l** were observed against any of those cultures previously treated with the same compounds compared to the MIC for the wild-type strain, a 16-fold increase in **Mnz** MIC was recorded against a *H. pylori* culture previously exposed to a 4-fold MIC concentration of this drug, which indicates that Mnz-resistant mutants have been generated. Accordingly, the ability of *H. pylori* to develop resistance to those **IV**-related compounds seems to be much less pronounced than that to develop resistance against **Mnz**.

### 3.4. Synergistic Interaction between Some ***IV***-Related Compounds and Rabeprazole

**Figure 6:**
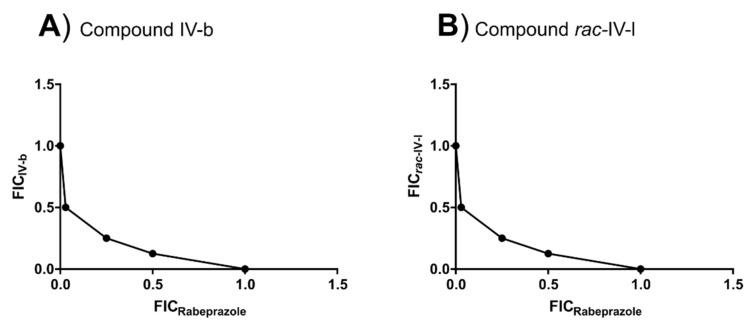
Synergism between rabeprazole and compounds **IV-b** (**A**) and ***rac*-IV-l** (**B**) against *H. pylori* strain ATCC 700392. The concave curve obtained in both graphs by representing FIC values indicates a synergistic relation between rabeprazole and those **IV**-related compounds. The closest point to the axes intersection relates to the most effective combination of compounds to inhibit *H. pylori* growth, which is recorded as FICI.

The bactericidal effect of compound **IV** and several derivatives in combination with anti-*H. pylori* drugs such as **Mnz**, **Cla**, omeprazole and rabeprazole was analysed in vitro, as well as that of compound **IV** combined with some of its derivatives. The synergistic effect was estimated by calculating the fractional inhibitory concentration index (FICI) for each pair of molecules. As the checkerboard assay requires previous knowledge of the MIC of the individual compounds, the MICs of omeprazole and rabeprazole against *H. pylori* strain ATCC 700,392 were also determined. Notably, the in vitro activity of rabeprazole (MIC = 0.5 μg/mL) was 64 times higher than that of omeprazole (MIC = 32 μg/mL). As seen in Table 4, whereas most compounds did not interact with **IV**, **Mnz**, **Cla**, omeprazole or rabeprazole (FICI > 0.5), compounds **IV-b** and ***rac*-IV-l** showed a synergistic relation (FICI = 0.5) with rabeprazole, indicating that the combined effect of these substances is significantly higher than the sum of their individual antimicrobial activities. Synergism was confirmed by plotting the FIC values of **IV-b** and ***rac*-IV-l** against the corresponding FIC values of rabeprazole as in both cases a concave curve was obtained (Figure 6). For those compounds, the most effective combinations for inhibiting *H. pylori* growth were 0.25 μg/mL of **IV-b** or 64 μg/mL of ***rac*-IV-l** plus 0.125 μg/mL of rabeprazole. Although significant synergism, as per our quantitative definition, was only found between the indicated pairs of substances, there were several compounds that clearly reduced the MICs of others. In particular, **IV-a** lowered the MIC of **IV** 64-fold (FIC = 0. 016; Table 4), whereas **IV** lowered that of **Mnz** 16-fold (FIC = 0.063). Notably, the combination of **Cla** with compounds **IV-d**, ***rac*-IV-j** or ***rac*-IV-l** reduced their MICs 8-fold (FIC = 0.125), an effect which was also observed for the omeprazole MIC when it was combined with **IV-b** (FIC = 0.125). On the other hand, the combination of rabeprazole with **IV-a** or **IV-d** lowered their MICs 16- and 8-fold (FIC = 0.0625 or 0.125, respectively). Thus, in conclusion, these compounds improved the in vitro anti-*H. pylori* activity of those other molecules and, remarkably, **IV** and **IV-b** increased, respectively, the antimicrobial effect of **Mnz** and omeprazole, two conventional drugs used to treat *H. pylori* infections.

## 4. Discussion

The emergence of resistant bacteria worldwide is a global health concern, of which the management requires coordinated efforts [43]. In particular, *H. pylori* resistance to currently used broad-spectrum antimicrobials has led to a decrease in the eradication rates, which brought about the inclusion of *H. pylori* in the first-ever list of antibiotic-resistant priority pathogens published by the WHO in 2017 [7]. In order to address this problem, new selective treatments have been proposed, one of them being the development of new compounds acting on specific bacterial targets such as flavodoxin [10]. This is an essential protein for *H. pylori* and it is absent in humans, which makes it a promising pharmacological target [11]. In earlier work, three flavodoxin inhibitors (**I**, **II** and **IV**) were discovered [16] and derivatised [17] to improve their therapeutic indexes against *H. pylori*. In the second round of optimization, the modification of nitro and sulphide groups present in one of the original leads (compound **IV**) increased its therapeutic activity against some reference strains and several drug-resistant clinical isolates. Moreover, this family of compounds was also able to decrease *H. pylori* gastric colonization rates in a mice model of infection and to eradicate it in up to 60% of mice treated [18]. The goal of the present study was to develop a better understanding of the antimicrobial spectrum and the mechanism of action of this family of antimicrobials. The compounds have been tested against 8 Gram-positive and 15 Gram-negative bacteria. The Gram-negative bacteria tested comprise 7 genera of the phylum *Proteobacteria*, of which the *Helicobacter* genus is represented by 3 enterohepatic species (*H. hepaticus*, *H. muridarum* and *H. bilis*) and 6 gastric species (*H. pylori*, *H. felis*, *H. suis*, *H. heilmannii*, *H. ailurogastricus* and *H. bizzozeronii*). Most IV-related compounds showed potent antimicrobial activity against *H. pylori*. However, the antimicrobial spectrum (Table 1) was not the same for all of them.

Compounds **IV** and ***rac*-IV-j** are extended-spectrum antimicrobials that are effective against the Gram-positive bacteria and Gram-negative bacteria of the *Helicobacter* genus (plus *C. jejuni*), but they are ineffective against any of the other Gram-negative bacteria tested, belonging to 5 different genera. As they are effective against potential pathogenic bacteria for humans [10], such as *H. pylori*, *H. suis,* *H. felis*, *H. heilmannii, H. bizzozeronii,* *C. jejuni,* some *Bacillus* species, *S. pneumoniae*, *E. faecalis, S. aureus* and *C. diphtheriae*, they could be used as co-adjuvants, or as an alternative treatment for the corresponding infections. This could be particularly useful for *C. jejuni*, *S. pneumoniae* and *S. aureus*, which were also included in the WHO priority list of antibiotic-resistant bacteria that represent a great threat to human health [7]. The additional compounds (**IV-a**, **IV-b**, **IV-c**, **IV-d** and ***rac*-IV-k**) are ineffective against Gram-positive and Gram-negative alike, except *H. pylori*, for which they all show a potent inhibitory activity (***rac*-IV-l,** perhaps related to its much high hydrophilicity, was ineffective against all bacterial species tested). Within the *Helicobacter* genus, we have determined that none of them inhibits the growth of enterohepatic species (Table 1), but their activity against other gastric species remains to be tested. These compounds (**IV-a**, **IV-b**, **IV-c**, **IV-d**, ***rac*-IV-k)** are thus narrow-spectrum antibiotics effective against *H. pylori* and perhaps against other gastric species of the *Helicobacter* genus, but not even against the enterohepatic *Helicobacter* species tested or *C. jejuni*. Importantly, these compounds are not effective against bacteria that may be part of the human microbiota, such as *E. faecalis*, *S. aureus*, *S. pneumoniae* and *E. coli* [10] at concentrations as high as 64 μg/mL. The narrow antimicrobial spectrum of these compounds may limit the side effects on microbiota of anti-*H. pylori* therapies based on or including them.

The mechanism of action of these antimicrobials is not fully understood. They were initially discovered as compounds that bind to and inhibit the in vitro activity of purified *Hp*-flavodoxin. All related inhibitors so far tested (this work and [16,17,18]) have been shown to bind to the purified protein. In this respect, most flavodoxins embed the FMN redox cofactor by sandwiching its isoalloxazine redox-active ring between two bulky residues (typically a tryptophan at the 50′ loop and a tyrosine at the 90′ loop). In contrast, at the equivalent position of the 50′ loop, *Hp*-Fld contains an alanine residue (A55), whose small size contributes to creating a distinct pocket at the si face of the cofactor. The flavodoxin inhibitors have been speculated to bind at that pocket [16]. As shown in Figure 5, this peculiar sequence feature is shared with the flavodoxins of gastric *Helicobacter* species, but is absent in those of enterohepatic species of this genus and in *C. jejuni*. Given the present lack of precise structural information about the inhibitors binding site, we have resourced to protein engineering to try to identify surface residues that may be at the binding site or nearby. Thus, we have mutated seven residues of the flavodoxin surface which are located either at a putative binding site identified through docking analysis (V113, Q115, T116 and K133A) or at the FMN binding site (A55 which is the residue that creates the pocket, and N14A and D142Y which interact with the phospho-ribityl moiety of the FMN cofactor). ITC analysis (Figure 4 and Figure S4, and Table 2) of the affinity of wild-type and mutant flavodoxins for two representative inhibitors (**IV** and **IV-c**) suggests that the residues located at the putative binding site do not interact with the inhibitors or lay close to the interacting site, as their replacement does not perturb the binding. The same appears to be the case of residues 14 and 142, in contact with the phosphate and ribityl moieties of the FMN cofactor. In contrast, the replacement of A55 by tryptophan stands out as the only replacement that clearly lowers the inhibitors’ affinity. The ITC analysis thus lends no support to the putative binding site suggested by the docking analysis, but is consistent with the A55 pocket being the likely binding site for the inhibitors, as previously proposed [16]. This observation will require further confirmation, as the A55W mutant weakens but does not abrogate the binding of the inhibitors.

Binding to the *Hp*-Fld specific pocket near the cofactor remains a likely structural explanation for the antimicrobial activity of the flavodoxin inhibitors and explains why compounds **IV-a**, **IV-b**, **IV-c**, **IV-d**, ***rac*-IV-k** are antimicrobials for *H. pylori*, but not for the three enterohepatic *Helicobacter* species tested, which lack the pocket. However, it does not explain why two inhibitors, **IV** and ***rac*-IV-j**, are effective not only for *H. pylori* but also for two enterohepatic *Helicobacter* species (*H. muridarum* and *H. bilis*)*,* for *C. jejuni* and, even more remarkably, for most Gram-positive bacteria tested. These two compounds, **IV** and ***rac*-IV-j**, unlike the other analogues, are the only nitro-derivatives of the tested series and it is possible that the nitro functionality allows them to act by an additional mechanism, which could explain their extended antimicrobial spectrum. As compounds **IV** and ***rac*-IV-j** are more cytotoxic towards HeLa cells [18] than the other compounds of this family, they might be targeting additional proteins or, once in the bacterial cells, they could be transformed into cytotoxic compounds, as has been described for **Mnz**, which also contains a nitro group. The greater permeability of monoderm Gram-positive bacteria compared to diderm Gram-negative ones might contribute to compounds **IV** and ***rac*-IV-j** reaching higher concentrations in the cytoplasm of these Gram-positive bacteria, where they could exert their alternative antimicrobial activity, unlike in Gram-negative bacteria. Whatever the case, it seems that replacing the nitro group in **IV** and ***rac*-IV-j** by its reduced amine version greatly increases the selectivity of this family of compounds, and turns them from extended-spectrum to narrow-spectrum antimicrobials highly specific to *H. pylori* and perhaps for other gastric *Helicobacter* species.

No spontaneous mutants were obtained when *H. pylori* cultures were incubated in the presence of high concentrations of some IV-related compounds under the same conditions, which allowed us to generate **Mnz** resistant mutants. As determined by Wang et al., **Mnz** showed an in vitro mutation rate of 6.9 × 10^−10^ per cell per division, which was less than those of **Cla**, ciprofloxacin, and rifampicin [44]. Given that all these antimicrobials are included in *H. pylori* eradication therapies [45,46,47,48,49,50], replacing some of them with **IV**-related compounds could help to reduce the development of drug resistance in this bacterium. On the other hand, the more severe antibiotic resistance associated with Gram-negative bacteria compared to Gram-positive ones appears to be related to several drug resistance mechanisms, including the overexpression of efflux pumps in the former [51,52,53]. Indeed, *H. pylori* multidrug-resistance is associated with the activation of efflux pumps [54,55]. Thus, compounds inhibiting efflux pumps are expected to influence internal concentrations of substances such as antibiotics, and combined use of EIs with antimicrobials might increase bacterial susceptibility [51,55,56,57]. CCCP, reserpine or valinomycin inhibit ABC, DMT, MATE, MFS and/or RND [51,55,58,59,60,61,62,63,64,65], which are the main conserved families of bacterial efflux pumps [51,65,66,67]. As the antimicrobial activity of compound **IV** derivatives is not greatly affected by those EIs, these **IV**-related antimicrobials do not seem to be substrates of the most common types of bacterial efflux pumps, which adds to their potential to bypass intrinsic resistance mechanisms of *H. pylori*.

The use of antimicrobial monotherapies might boost the selection of drug-resistant strains, so combinations of drugs could be useful to reduce the selection of antibiotic resistance [37,38,68]. In this regard, the interaction between **IV**-related compounds and other pharmacological entities in use against *H. pylori* has been evaluated by the checkerboard assay [69]. Most of the derivatives tested did not exhibit synergy when combined with lead compound **IV**, **Mnz**, **Cla**, omeprazole or rabeprazole. However, compounds **IV-b** and ***rac*-IV-l** showed synergy with rabeprazole. While both omeprazole and rabeprazol inhibit urease activity in vitro, rabeprazol does it at a lower concentration [70,71], which agrees with our determined MICs for either PPI. Rabeprazole effect on gastric acid secretion seems to be more potent and fast than that of omeprazole [71] and it appears to be less affected by CYP2C19 polymorphisms [45]. These facts, together with the synergy observed, makes rabeprazole the PPI of choice in a treatment based on **IV**-related compounds. Since two compounds that show a synergistic relationship are not expected to act on the same target or, at least, on the same region of the target, the human gastric proton pump (H^+^/K^+^-ATPase) and *H. pylori* urease might be discarded as targets of compounds **IV-b** and ***rac*-IV-l** [71]. Importantly, even if synergism was not reached, compounds **IV** and **IV-b** decreased 8- and 16-fold, respectively, the MICs of omeprazole and **Mnz** against *H. pylori*, which could enhance their activities in a combinatory therapy, and supports the potential of compounds **IV** and **IV-b** as adjuvants in current combinatory therapies containing **Mnz** or omeprazole.

## 5. Conclusions

Compound **IV** and related inhibitors of the flavodoxin from *Helicobacter pylori* are effective antimicrobials against different bacteria. Those bearing an amine functionality are narrow-spectrum antimicrobials highly specific against *H. pylori* and, possibly, other gastric, but not enterohepatic, *Helicobacter* species. The two compounds tested that contain a nitro functionality show an extended-spectrum activity against Gram-positive bacteria, the *Helicobacter* genus and *C. jejuni*. The extended spectrum antimicrobials may find use in novel therapies against Gram-positive bacteria, while the narrow-spectrum ones may be useful against *H. pylori.* Interestingly, these narrow spectrum antimicrobials are not substrates of common efflux pumps, appear to have a lower resistance rate than **Mnz,** show synergy with rabeprazole and may contribute to lowering the development of drug resistance in *H. pylori*. Besides, they might help to reduce the damage to the microbiota if included in eradication therapies, replacing some of the currently used antimicrobials.

## Figures and Tables

**Table 1:** In vitro activity of IV-related compounds against several bacteria from different phyla.

Gram	Bacterial Species (Strain)	Compound MIC (μg/mL)									
		IV	IV-a	IV-b	IV-c	IV-d	*rac*- IV-j	*rac*- IV-k	*rac*-IV-l	Mnz	Cla
-	*H. pylori* (ATCC 700392) *	2	8	1	2	8	1	16	256	2	≤0.032
-	*H. felis* (JKM5)	8					32				≤0.031
-	*H. suis* (HS1)	4					4				0.25
	(HS5)	4					2				≤0.031
-	*H. heilmannii* (ASB1.4)	2					2				0.125
	(ASB2)	2					1				≤0.031
-	*H. ailurogastricus * (ASB7)	4					1				0.125
	(ASB9)	4					4				0.5
-	*H. bizzozeronii* (ASB22 kol15)	16					16				≤0.031
	(10)	8					32				≤0.031
	(Heydar)	8					32				≤0.031
-	*H. hepaticus* (ATCC 51449/3B1)	64	>64	>64	>64	>64	64	>64	>64	>64	0.063
-	*H. muridarum*	1	>64	>64	>64	>64	4	>64	>64	>64	>64
-	*H. bilis*	16	>64	>64	>64	>64	16	>64	>64	>64	4
-	*C. jejuni* (ATCC 33560) *	2	>64	>64	>64	>64	4	>64	>64	1	4
-	*S.* Typhimurium (SV 5015) *	>64	>64	>64	>64	>64	>64	>64	>64	>64	>64
-	*E. coli* (ATCC 10536) *	>64	>64	>64	>64	>64	>64	>64	>64	>64	16
-	*P. aeruginosa* (ATCC 15442)	>64	>64	>64	>64	>64	>64	>64	>64	>64	8
	(MPAO1)	>512	>512	>512	>512	>512					
	(PW9682)	>512	>512	>512	>512	>512					
-	*S. maltophilia* K279a	>512	>512	>512	>512	>512					
	(K279a Δ*rmlBACD*)	>512	>512	>512	>512	>512					
-	*K. pneumoniae* 3025	>512	>512	>512	>512	>512					
	(CWK43)	>512	>512	>512	>512	>512					
+	*Bacillus* sp. (CECT 40)	4	>64	>64	>64	>64	4	>64	>64	>64	0.063
+	*S. pneumoniae* (ATCC 49619)	8	>64	>64	64	>64	16	>64	>64	>64	≤0.032
+	*L. monocytogenes* (ATCCBAA-679)	>64	>64	>64	>64	>64	>64	>64	>64	>64	0.25
+	*E. faecalis* (JH2-2)	2	>64	>64	>64	>64	4	>64	>64	>64	0.25
+	*S. aureus* (ATCC 29213)	16	>64	>64	>64	>64	8	>64	>64	>64	≤0.032
	BB pH 5	16					16–64				8–16
	MH broth pH 7	16					16				0.5–1
+	*C. diphtheriae* (ATCC 39255)	16	64	>64	8	>64	32	>64	>64	>64	0.063
+	*C. ammoniagenes* (ATCC 7862)	16	>64	>64	>64	>64	16	>64	>64	>64	≤0.032
+	*M. smegmatis* (ATCC 700084)	>64	>64	>64	>64	>64	>64	>64	>64	>64	4

* Flavodoxins that appear described as essential in the DEG database [40]. Not being in the DEG database does not necessarily mean they are essential.

**Table 2:** Thermodynamic parameters of the complexes formed between wild-type and several mutant flavodoxins with compounds **IV** and **IV-c** ^a^.

Flavodoxin Variant	IV				IV-c			
	*K_d_*^b^ (μM)	Δ*G* ^c^ (kcal/mol)	Δ*H* ^d^ (kcal/mol)	−*T*Δ*S* ^e^ (kcal/mol)	*K_d_*^b^ (μM)	Δ*G* ^c^ (kcal/mol)	Δ*H* ^d^ (kcal/mol)	−*T*Δ*S* ^e^ (kcal/mol)
WT	0.48	−8.58	3.77	−12.35	0.67	−8.39	3.10	−11.49
V113W	0.50	−8.56	3.69	−12.25	1.45	−7.93	1.40	−9.33
Q115W	0.52	−8.54	3.29	−11.83	0.69	−8.37	2.36	−10.73
T116W	1.10	−8.10	2.58	−10.68	1.43	−7.94	1.68	−9.62
K133A	0.85	−8.25	2.38	−10.63	0.77	−8.31	2.10	−10.41
N14A	0.45	−8.62	2.10	−10.72	1.76	−7.82	2.61	−10.43
A55W	2.33	−7.65	4.50	−12.15	1.05	−8.12	1.70	−9.82
D142Y	1.18	−8.05	1.13	−9.18	0.44	−8.64	0.92	−9.56
		−8.29 ± 0.35 Mean ± SD				−8.19 ± 0.28 Mean ± SD		

^a^ Obtained from calorimetric titrations in 50 mM EPPS, pH 9. ^b^ Relative error in *K_d_* is 10%. ^c^ Calculation of Gibbs energy change was based on Δ*G* = *RT*ln*K_d_*. Absolute error in Δ*G* is 0.1 kcal/mol. ^d^ Absolute error in Δ*H* is 0.3 kcal/mol. ^e^ Entropic contribution was calculated according to: −*T*Δ*S* = Δ*G* − Δ*H*. Absolute error in −*T*Δ*S* is 0.3 kcal/mol [41].

**Table 3:** In vitro activity of IV-related compounds alone and in combination with CCCP, reserpine and valinomycin against *H. pylori* strain ATCC 700392.

Compound	MIC ^a^ (μg/mL)			
	Without any EI	CCCP	Reserpine	Valinomycin
**IV**	2	0.5	0.5	0.5
**IV-a**	8	8	4	4
**IV-b**	1	2	2	2
**IV-c**	2	4	4	4
**IV-d**	8	32	32	32
***rac*-IV-j**	1	4	2	2
***rac*-IV-k**	16	16	16	8
***rac*-IV-l**	>64	>64	>64	>64
**Mnz**	2	4	4	2
**Cla**	≤0.032	≤0.032	≤0.032	≤0.032

^a^ The MIC values were assayed in the presence of non-lethal concentrations of the following EPIs: CCCP (0.08 μg/mL), reserpine (3.12 μg/mL) and valinomycin (2.50 μg/mL).

**Table 4:** FICs and FICIs of IV-related compounds in combination with either **IV**, **Mnz**, **Cla**, omeprazole or rabeprazole ^a^.

Combination Compound	IV-Related Compound	FIC_combination compound_	FIC_IV-related compound_	FICI ^b^
	**IV-a**	0.016	0.5	0.516
	**IV-b**	1	1	2
	**IV-c**	1	1	2
**IV**	**IV-d**	1	1	2
	***rac*-IV-j**	0.25	0.5	0.75
	***rac*-IV-k**	1	1	2
	***rac*-IV-l**	0.25	0.5	0.75
	**IV**	0.063	0.5	0.56
	**IV-a**	0.5	0.25	0.75
	**IV-b**	0.5	0.5	1
**Mnz**	**IV-c**	0.5	0.5	1
	**IV-d**	0.5	0.5	1
	***rac*-IV-j**	0.5	0.5	1
	***rac*-IV-k**	0.5	0.5	1
	***rac*-IV-l**	0.5	0.5	1
	**IV**	0.5	0.5	1
	**IV-a**	0.5	0.25	0.75
	**IV-b**	0.5	0.5	1
**Cla**	**IV-c**	0.5	0.25	0.75
	**IV-d**	0.5	0.125	0.62
	***rac*-IV-j**	0.5	0.125	0.62
	***rac*-IV-k**	0.25	0.5	0.75
	***rac*-IV-l**	0.5	0.125	0.62
	**IV**	0.5	0.5	1
	**IV-a**	0.25	0.5	0.75
	**IV-b**	0.125	0.5	0.62
Omeprazole	**IV-d**	0.25	0.5	0.75
	***rac*-IV-j**	0.5	0.5	1
	***rac*-IV-k**	0.25	0.5	0.75
	***rac*-IV-l**	0.5	0.5	1
	**IV**	1	1	2
	**IV-a**	0.5	0.063	0.56
	**IV-b**	0.25	0.25	0.5
Rabeprazole	**IV-d**	0.5	0.125	0.62
	***rac*-IV-j**	0.5	0.5	1
	***rac*-IV-k**	0.5	0.5	1
	***rac*-IV-l**	0.25	0.25	0.5

^a^ Values were determined against *H. pylori* ATCC 700392. ^b^ The fractional inhibitory index (FICI) was calculated as follows: FICI = (MIC_Combination compound in the presence of **IV**-related compound_/MIC_Combination compound alone_) + (MIC_**IV**-related compound in the presence of Combination compound_/MIC_**IV**-related compound alone_). A FICI value of ≤0.5 indicates synergy, a value from 0.5 to 4 denotes no interaction and a value of >4 expresses antagonism.

## Data Availability

The data to this study can be shared upon reasonable request from the corresponding author.

## Supplementary Materials

The following are available online at https://www.mdpi.com/article/10.3390/ijms221810137/s1.
