# Supplementary material for: Selective Targeting of Human and Animal Pathogens of the Helicobacter Genus by Flavodoxin Inhibitors: Efficacy, Synergy, Resistance and Mechanistic Studies
Source: Int J Mol Sci. 2021 Sep 20;22(18):10137. doi: 10.3390/ijms221810137 (PMC8467567; doi:10.3390/ijms221810137)
Supplement: Supplementary file 1 [file ijms-22-10137-s001.zip › ijms-1338710-supplementary.pdf]

Selective targeting of human and animal pathogens of the *Helicobacter* genus by flavodoxin inhibitors: efficacy, synergy, resistance, and mechanistic studies

Sandra Salillas[1,2,3], Juan José Galano-Frutos[1,2,3], Alejandro Mahía[1,2,3], Ritwik Maity[1,2,3], María Conde-Giménez[1,2,3], Ernesto Anoz-Carbonell[1,2,4], Helena Berlamont[5], Adrián Velazquez-Campoy[1,2,3,6,7], Eliette Touati[8], Uwe Mamat[9], Ulrich E. Schaible[9], José A. Gálvez[10], María D. Díaz de Villegas[10], Freddy Haesebrouck[5], José A. Aínsa[1,3,4,11], Javier Sancho[1,2,3*]

[1]Biocomputation and Complex Systems Physics Institute (BIFI)-Joint Units: BIFI-IQFR (CSIC) and GBsC-CSIC, University of Zaragoza, Zaragoza 50018, Spain

[2]Departamento de Bioquímica y Biología Molecular y Celular, Faculty of Science, University of Zaragoza, Zaragoza 50009, Spain

[3]Aragon Health Research Institute (IIS Aragón), Zaragoza 50009, Spain

[4]Departamento de Microbiología, Pediatría, Radiología y Salud Pública, Faculty of Medicine, University of Zaragoza, Zaragoza 50009, Spain

[5]Department of Pathobiology, Pharmacology and Zoological Medicine, Faculty of Veterinary Medicine, Ghent University, Salisburylaan 133, B9820 Merelbeke, Belgium

[6]ARAID Foundation, Government of Aragon, 50018 Zaragoza, Spain

[7]CIBER de Enfermedades Hepáticas y Digestivas CIBERehd, Instituto de Salud Carlos III, Madrid 28029, Spain

[8] Unit of Helicobacter Pathogenesis, CNRS UMR2001, Department of Microbiology, Institut Pasteur, 25-28 Rue du Dr. Roux, Paris 75724, France

[9] Cellular Microbiology, Program Area Infections, Research Center Borstel, Leibniz Lung Center, 23845 Borstel, Germany.

[10]Instituto de Síntesis Química y Catálisis Homogénea (ISQCH), CSIC - Departamento de Química Orgánica, Faculty of Science, University of Zaragoza, Zaragoza 50009, Spain

[11]CIBER de Enfermedades Respiratorias–CIBERES, Instituto de Salud Carlos III, Madrid 28029, Spain

*Correspondence to: jsancho@unizar.es

Supplementary files

Figure S1

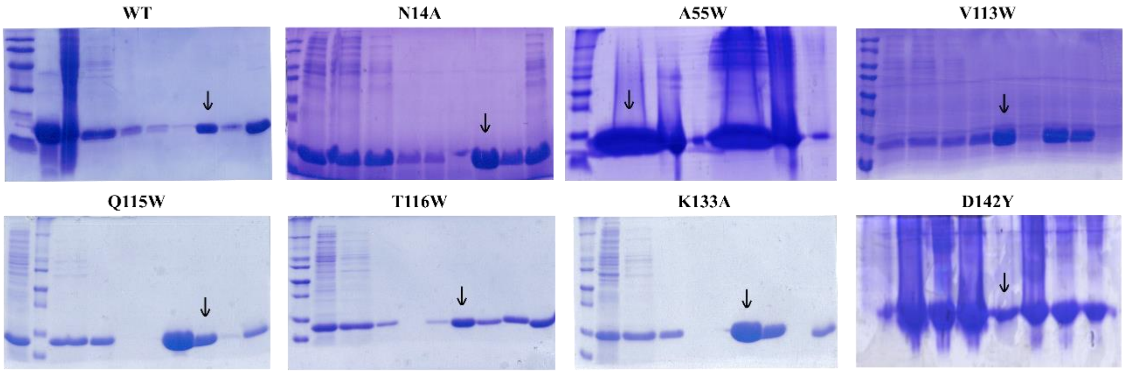

SDS_PAGE corresponding to the purification of the different flavodoxin variants. Arrows indicate the final samples used for further analysis.

Figure S2

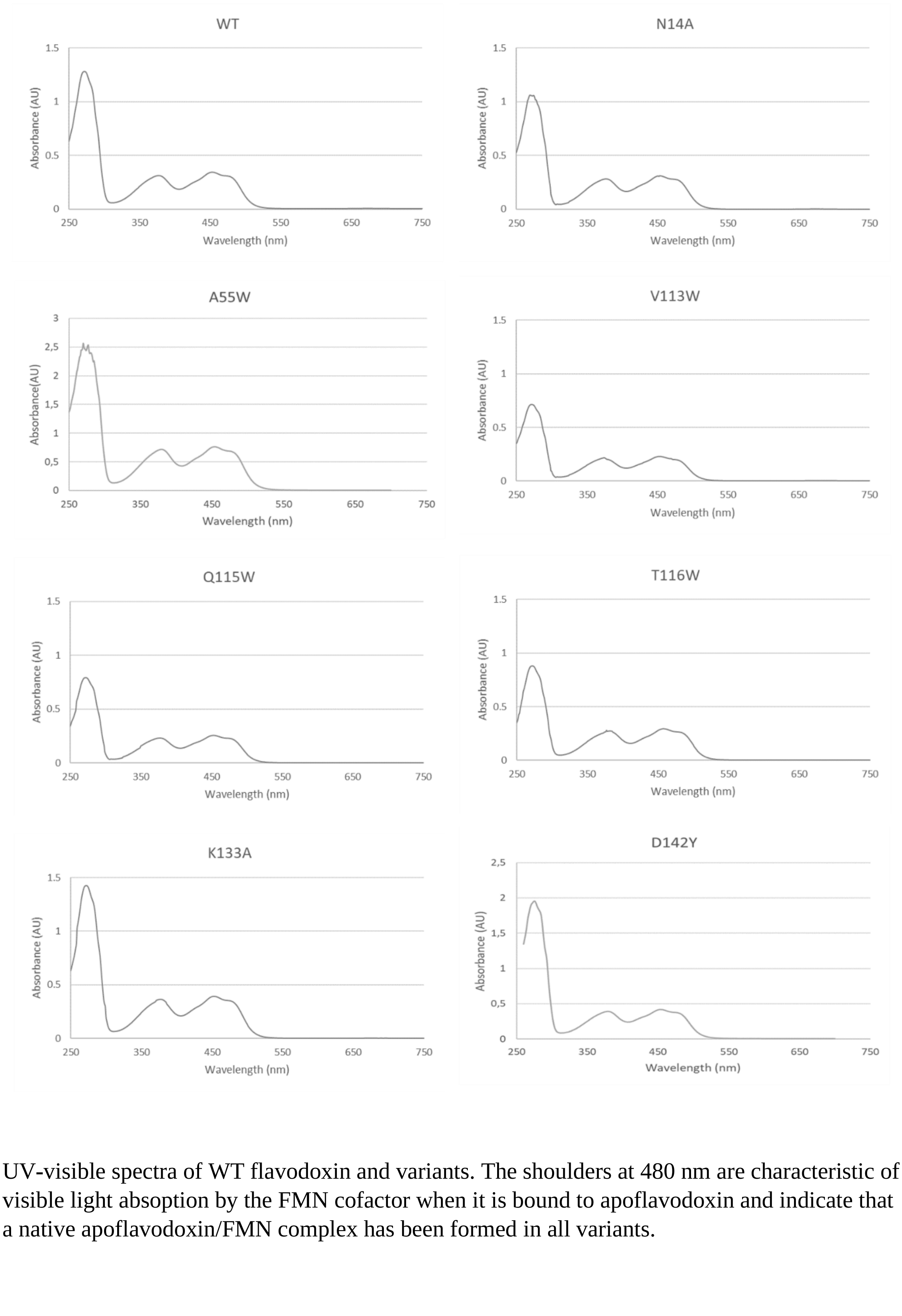

UV-visible spectra of WT flavodoxin and variants. The shoulders at 480 nm are characteristic of visible light absoption by the FMN cofactor when it is bound to apoflavodoxin and indicate that a native apoflavodoxin/FMN complex has been formed in all variants.

Figure S3

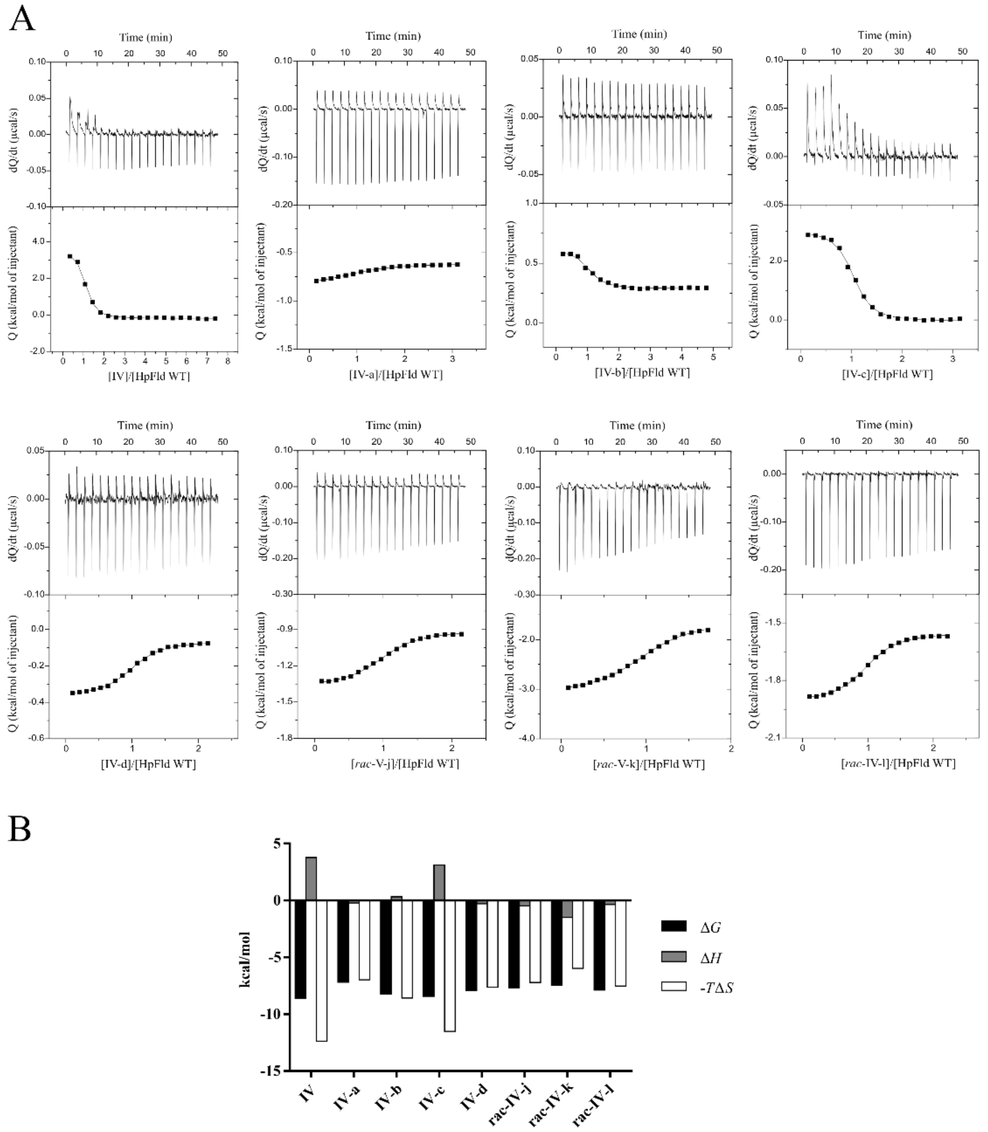

Thermodynamic analysis of the interaction between wild-type *Hp*-Fld and IV-related compounds by ITC. (A) Calorimetric titrations for compounds **IV**, **IV-a**, **IV-b**, **IV-c**, **IV-d**, ***rac*-IV-j**, ***rac*-IV-k** and ***rac*-IV-l**. The upper plots show the thermograms (thermal power as a function of time), whereas the lower panels display the binding isotherms (titrant-normalised heat effects as a function of the ligand:protein molar ratio in the cell), the solid lines corresponding to the best fits. (B) Thermodynamic parameters of each flavodoxin-compound interaction (see also Table 2). Gibbs energy (ΔG), enthalpy (ΔH) and entropic contribution ($-T\Delta S$) are represented in black, grey and white bars, respectively.

Figure S4

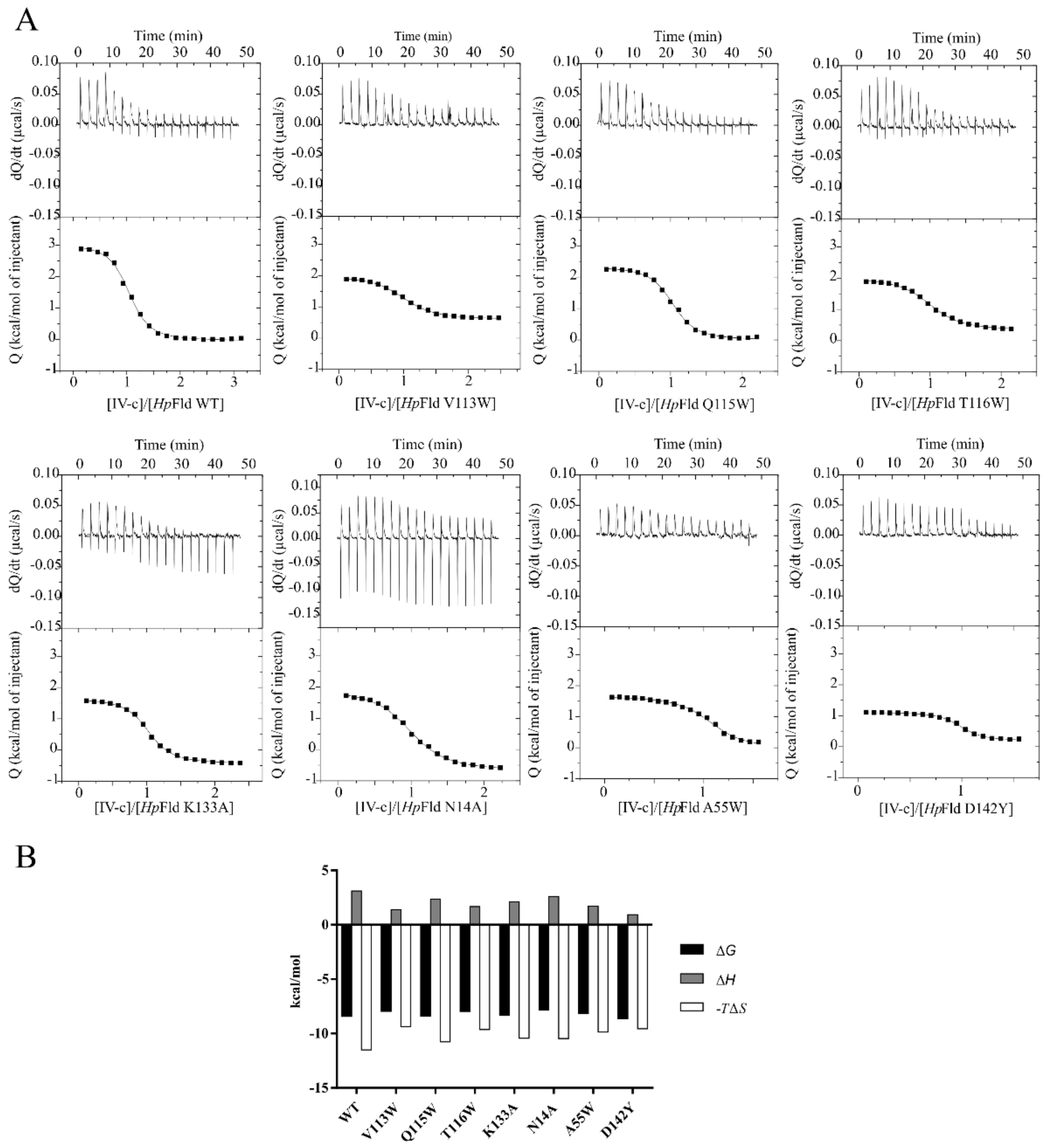

Thermodynamic analysis of the interaction between compound **IV-c** and wild-type *Hp*-Fld and mutants V113W, Q115W, T116W, K133A, N14A, A55W and D142Y by ITC. (A) The upper plots show the thermograms (thermal power as a function of time), whereas the lower panels show the binding isotherms (normalised heats as a function of the ligand:protein molar ratio). In the latter, the solid line corresponds to the best fit. (B) Thermodynamic parameters of each flavodoxin-compound interaction. Gibbs energy (ΔG), enthalpy (ΔH) and entropic contribution ($-T\Delta S$) are represented in black, grey and white bars, respectively**.**

Table S1. Thermodynamic parameters for the interaction of wild-type *Hp*-Fld with **IV**-related compounds[a.]

Compound	***K_d*** [b] ***(μM)***	***ΔG*** [c] ***(kcal/mol)***	***ΔH*** [d] ***(kcal/mol)***	***-TΔS*** [e] ***(kcal/mol)***
IV	0.48	-8.62	3.77	-12.38
IV-a	5.26	-7.20	-0.23	-6.97
IV-b	0.91	-8.24	0.33	-8.57
IV-c	0.67	-8.42	3.10	-11.51
IV-d	1.55	-7.92	-0.30	-7.62
rac-IV-j	2.33	-7.68	-0.47	-7.21
rac-IV-k	3.36	-7.46	-1.49	-5.98
rac-IV-l	1.65	-7.88	-0.36	-7.52

[a]Obtained from calorimetric titrations in 50 mM EPPS, pH 9 (for **IV**, **IV-a**, **IV-b**, **IV-c**, ***rac*-IV-j**, ***rac*-IV-k** and ***rac*-IV-l**) or 50 mM Tris, pH 9 (for **IV-d**). [b]Relative error in K_d is 10%. [c] Calculation of Gibbs energy change was based on $\Delta G = RT\ln K_d$. Absolute error in ΔG is 0.1 kcal/mol. [d]Absolute error in ΔH is 0.3 kcal/mol. [e]Entropic contribution was calculated according to: $-T\Delta S = \Delta G - \Delta H$. Absolute error in $-T\Delta S$ is 0.3 kcal/mol.[40] .

Table S2. Characteristics of bacteria used to study antimicrobial activity of IV-related compounds

	**Phylum**	**Gram**	**Flavodoxin**[a]	**Residue at position 55**[c]
H. pylori	Proteobacteria	Negative	Long	Ala
H. felis	Proteobacteria	Negative	Long	Ala
H. hepaticus	Proteobacteria	Negative	Long	Tyr
H. muridarum	Proteobacteria	Negative	Long	Trp
H. bilis	Proteobacteria	Negative	Long	Trp
H. suis	Proteobacteria	Negative	Long	Ala
H. heilmannii	Proteobacteria	Negative	Long	Ala
H. ailurogastricus	Proteobacteria	Negative	Long	Ala
H. bizzozeronii	Proteobacteria	Negative	Long	Ala
C. jejuni	Proteobacteria	Negative	Long	Trp
S. Typhimurium	Proteobacteria	Negative	Long	Trp
E. coli	Proteobacteria	Negative	Long and short	Trp / Thr / His
P. aeruginosa	Proteobacteria	Negative	Long and short	Thr
S. maltophilia	Proteobacteria	Negative	Long and short	Asp
K. pneumoniae	Proteobacteria	Negative	Long	Trp / Leu
Bacillus sp.	Firmicutes	Positive	Short	Trp[b]
S. pneumoniae	Firmicutes	Positive	Short	Tyr
L. monocytogenes	Firmicutes	Positive	Short	Val
E. faecalis	Firmicutes	Positive	No	-
S. aureus	Firmicutes	Positive	No	-
C. diphtheriae	Actinobacteria	Positive	No	-
C. ammoniagenes	Actinobacteria	Positive	No	-
M. smegmatis	Actinobacteria	Positive	No	-

[a]According to UniProt and [10,71].
[b]For *B. subtilis*.
[c]According to *Hp*-Fld sequence.

^{1}H-NMR and ^{13}C-NMR spectra of the synthesised compounds

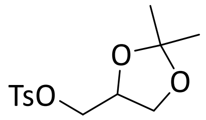

***rac*-S1**

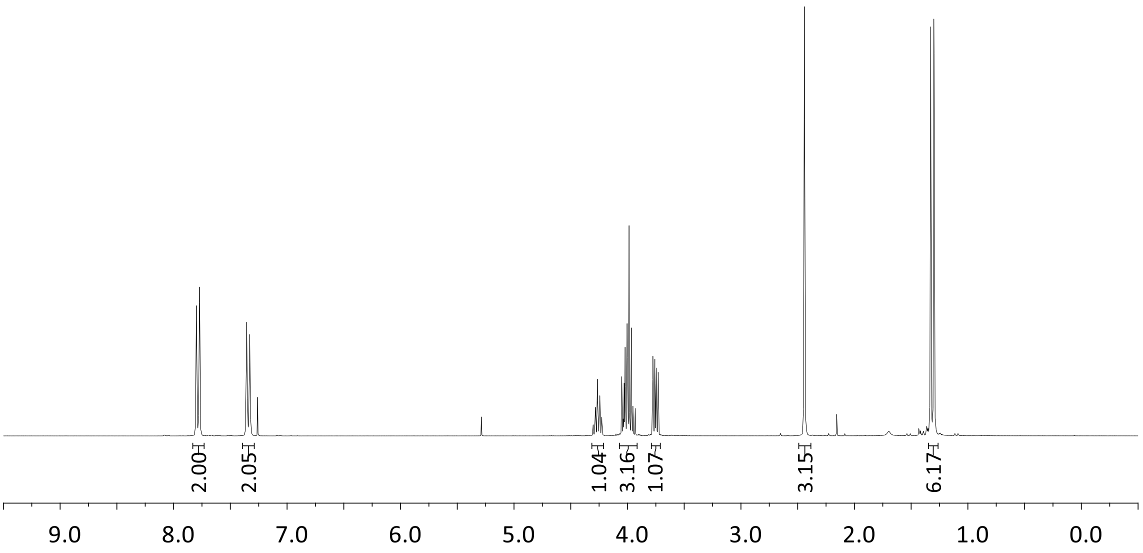

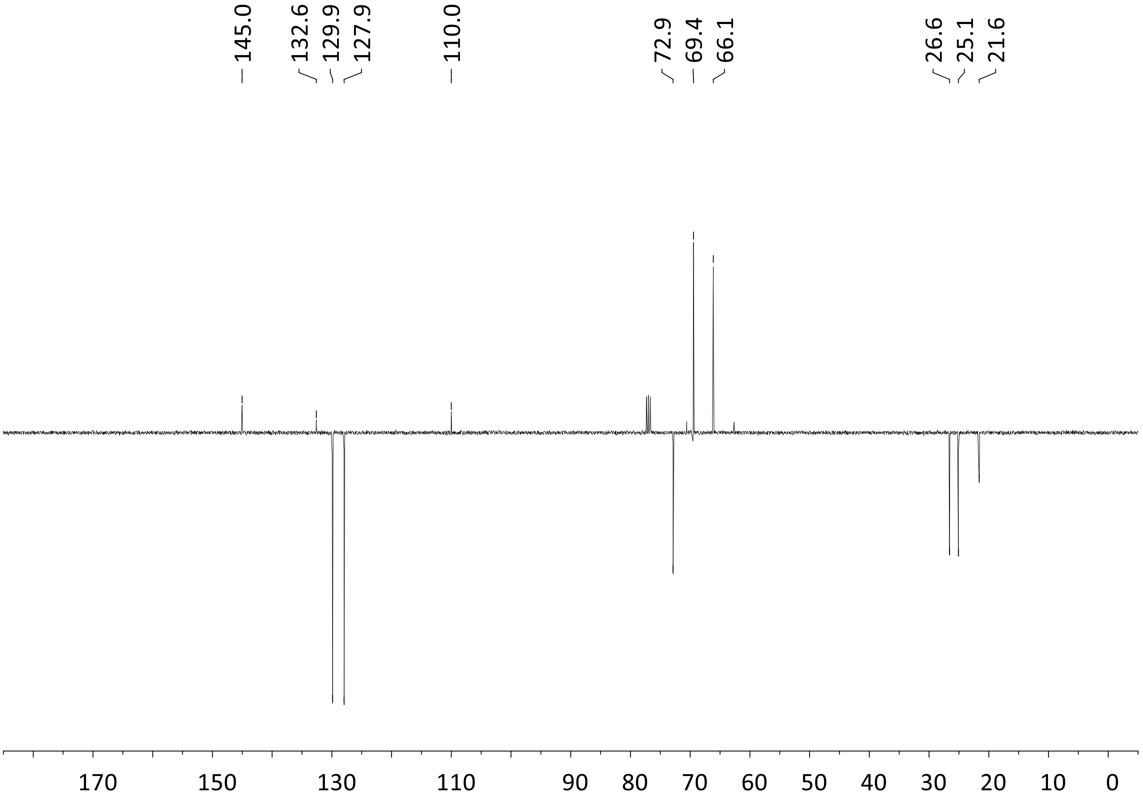

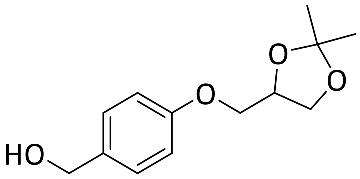
HO
O
O
O

***rac*-S2**

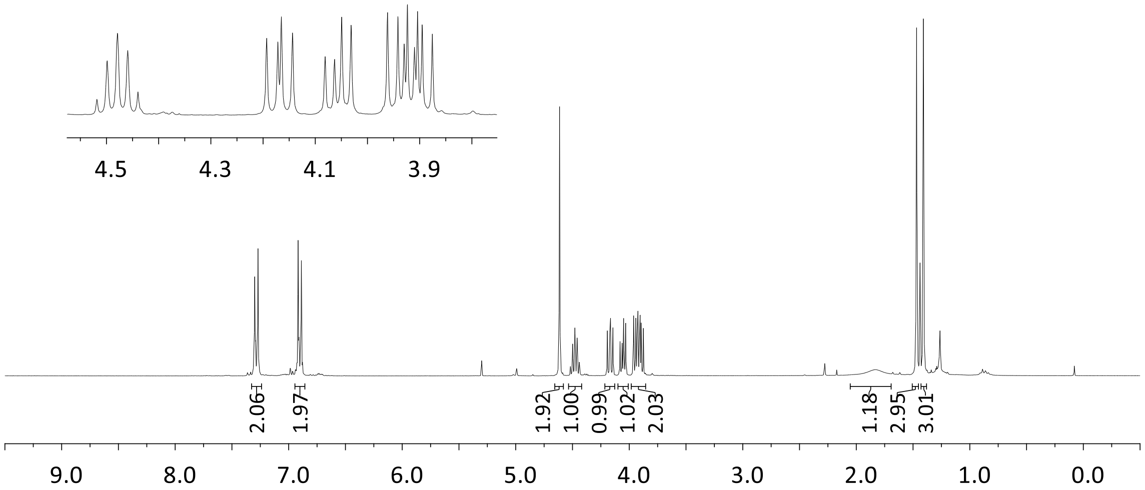
4.5
4.3
4.1
3.9
2.06
1.97
1.92
1.00
0.99
1.02
2.03
1.18
2.95
3.01
9.0
8.0
7.0
6.0
5.0
4.0
3.0
2.0
1.0
0.0

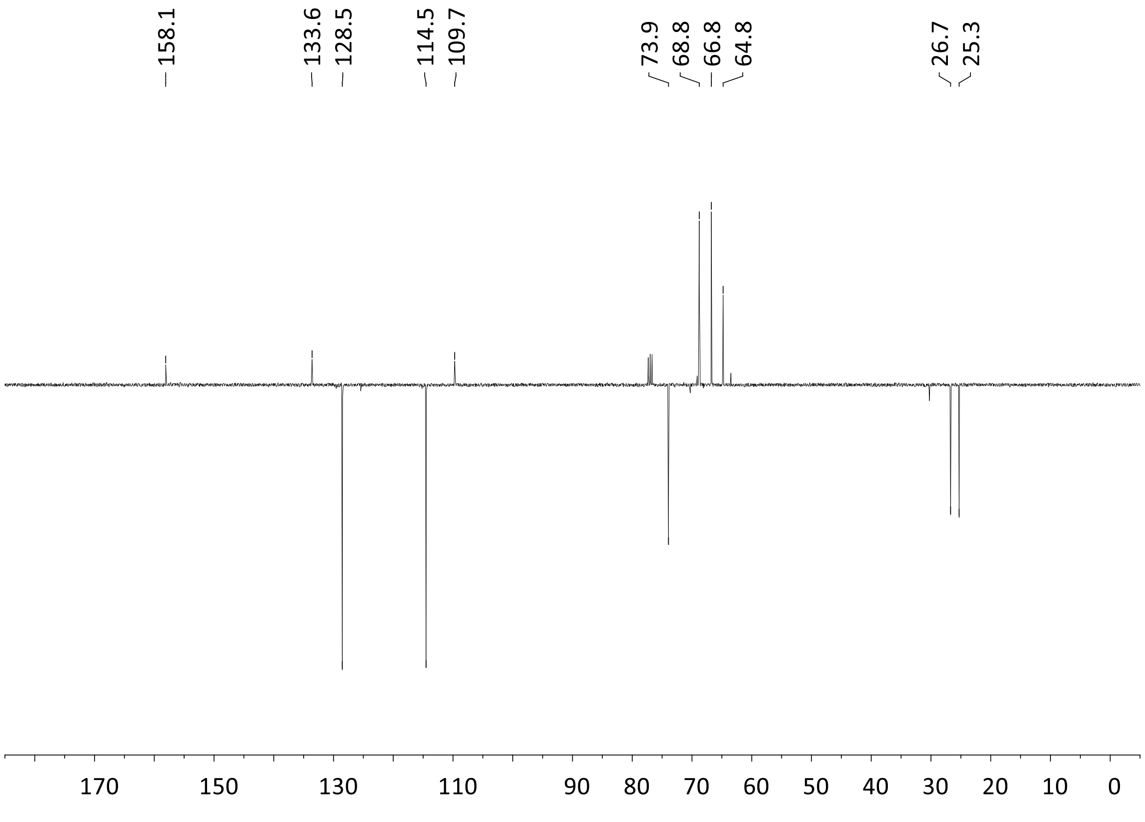
158.1
133.6
128.5
114.5
109.7
73.9
68.8
66.8
64.8
26.7
25.3
170
150
130
110
90
80
70
60
50
40
30
20
10
0

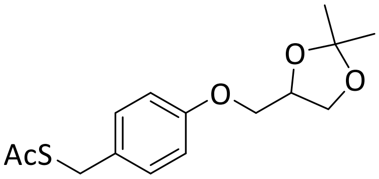
AcS
O
O
O

***rac*-S3**

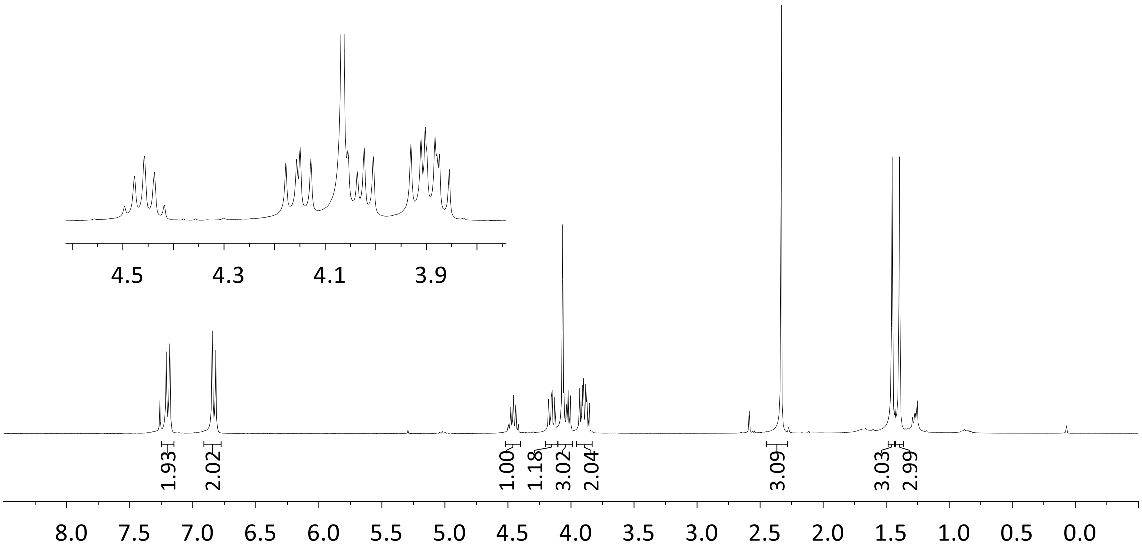
4.5
4.3
4.1
3.9
1.93
2.02
1.00
1.18
3.02
2.04
3.09
3.03
2.99
8.0 7.5 7.0 6.5 6.0 5.5 5.0 4.5 4.0 3.5 3.0 2.5 2.0 1.5 1.0 0.5 0.0

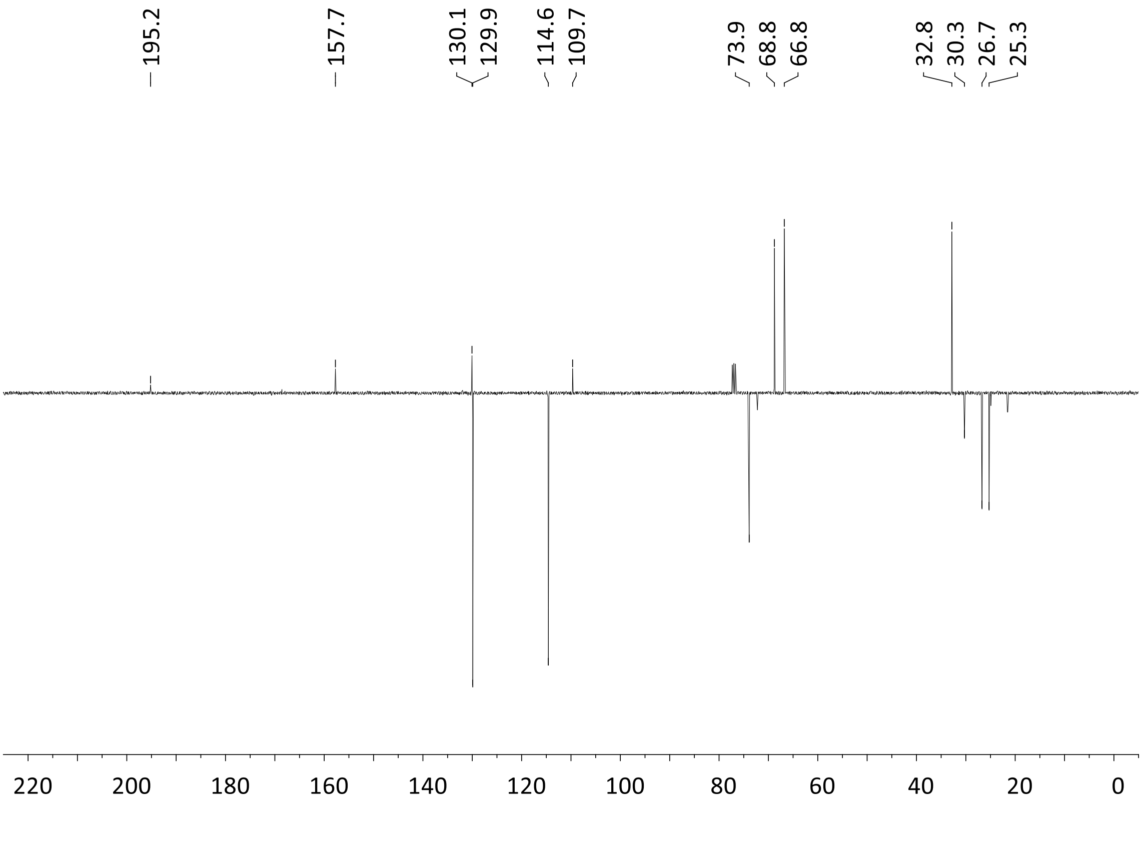
195.2
157.7
130.1
129.9
114.6
109.7
73.9
68.8
66.8
32.8
30.3
26.7
25.3
220 200 180 160 140 120 100 80 60 40 20 0

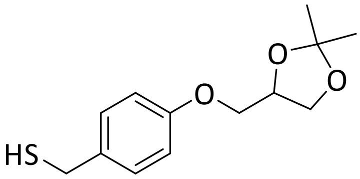

HS
O
O
O

***rac*-S4**

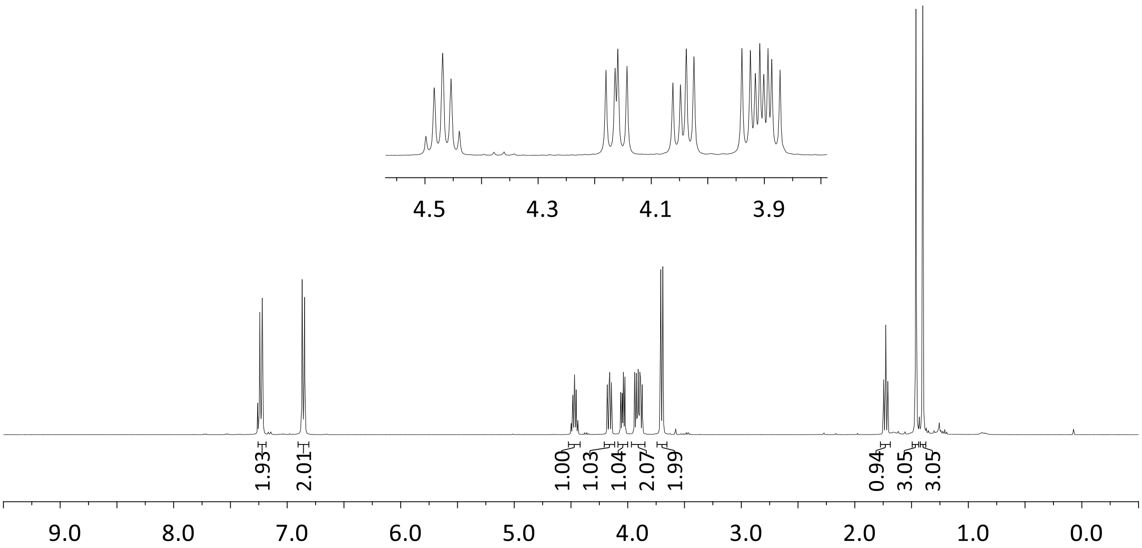

4.5
4.3
4.1
3.9
1.93
2.01
1.00
1.03
1.04
2.07
1.99
0.94
3.05
3.05
9.0
8.0
7.0
6.0
5.0
4.0
3.0
2.0
1.0
0.0

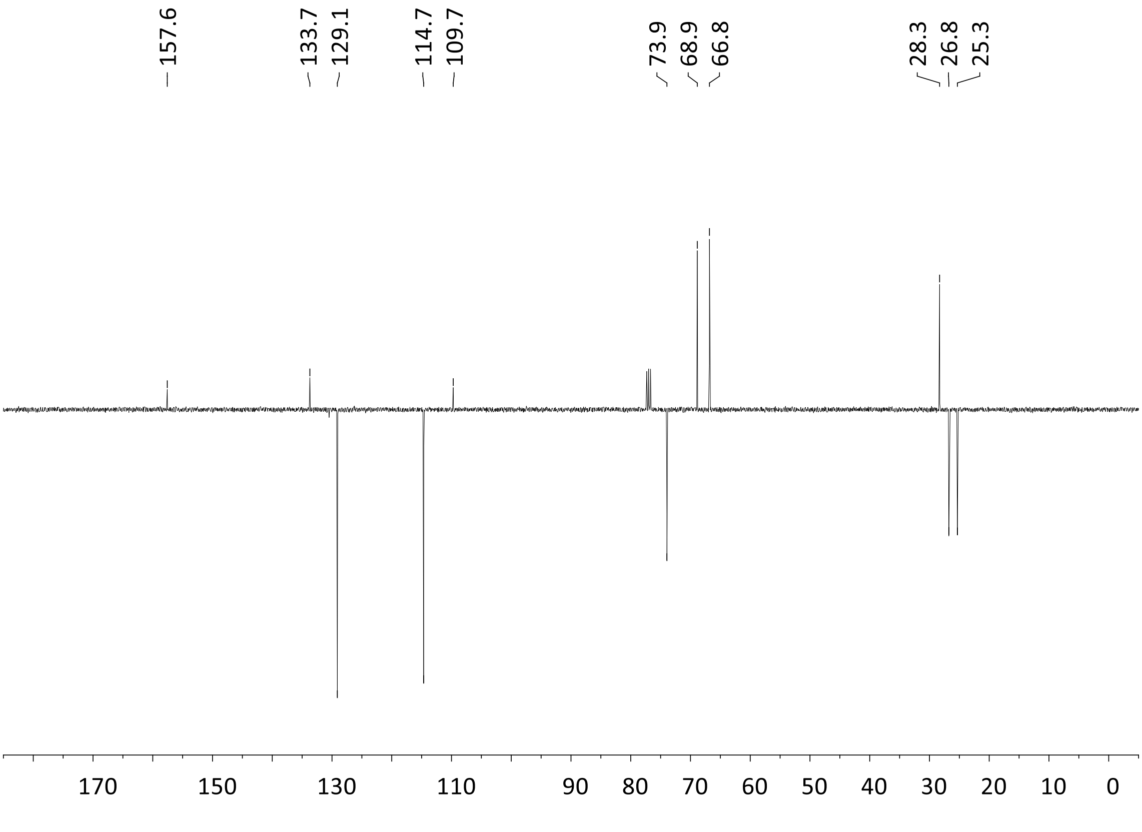

157.6
133.7
129.1
114.7
109.7
73.9
68.9
66.8
28.3
26.8
25.3
170
150
130
110
90
80
70
60
50
40
30
20
10
0

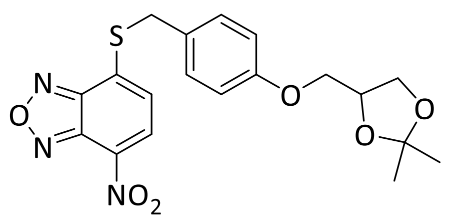

***rac*-S5**

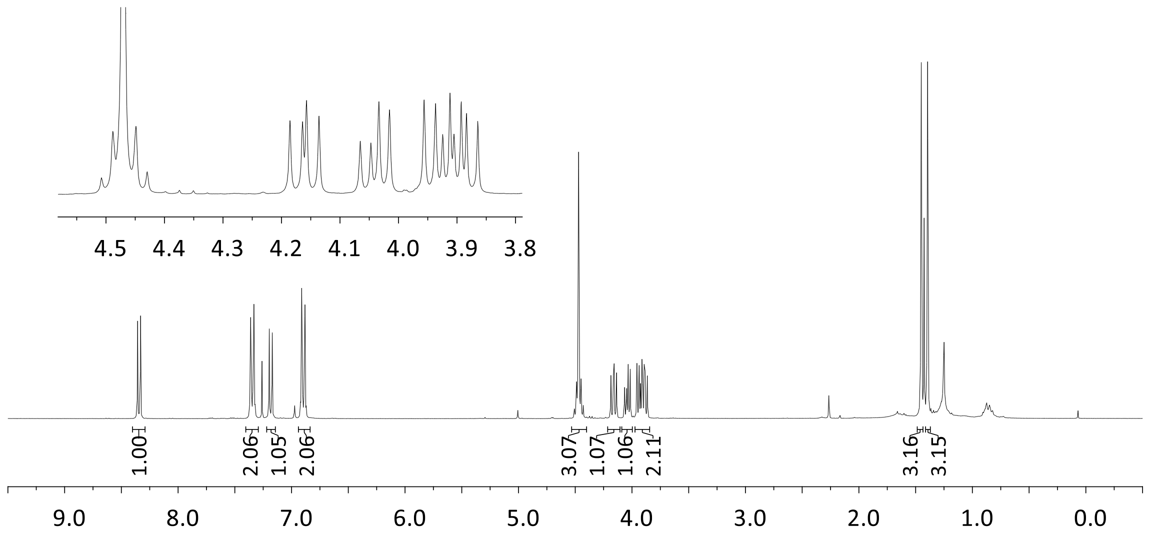

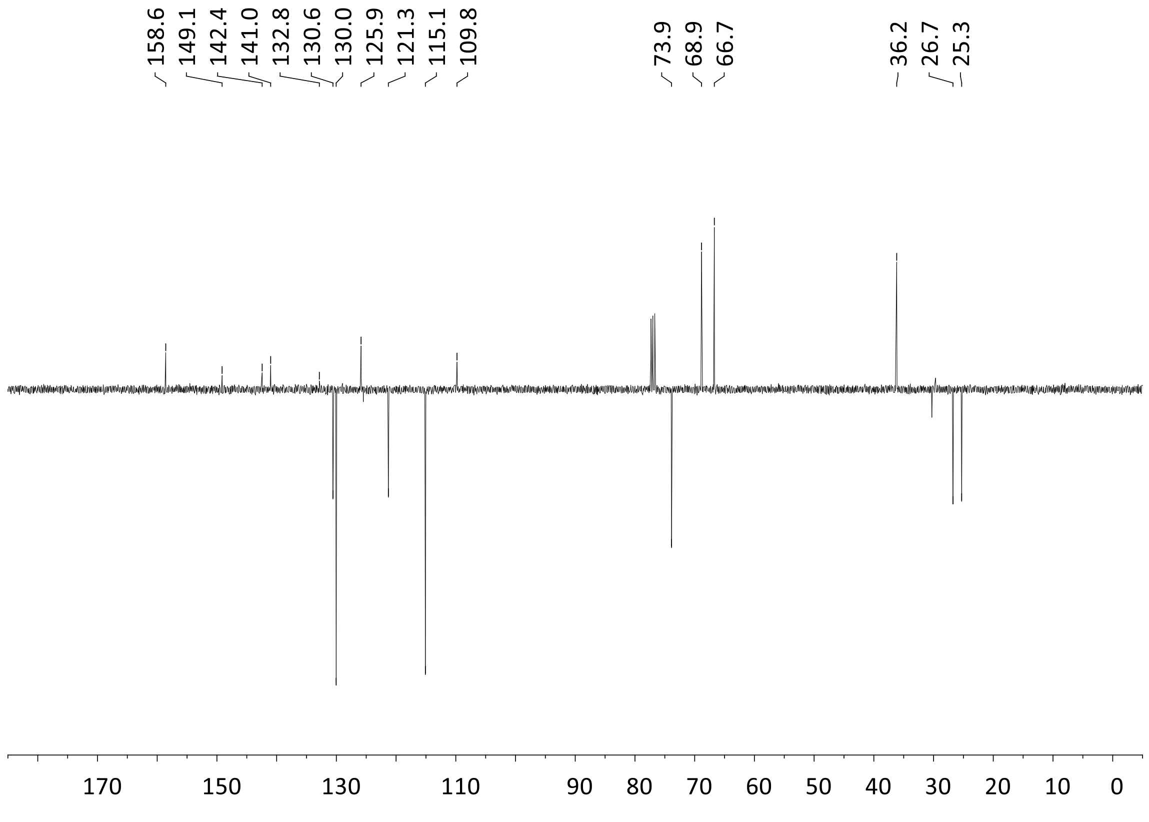

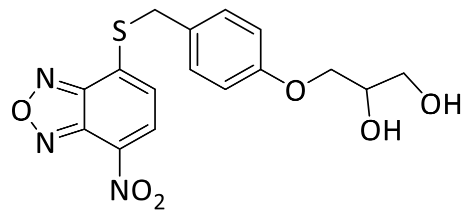

***rac*-IV-j**

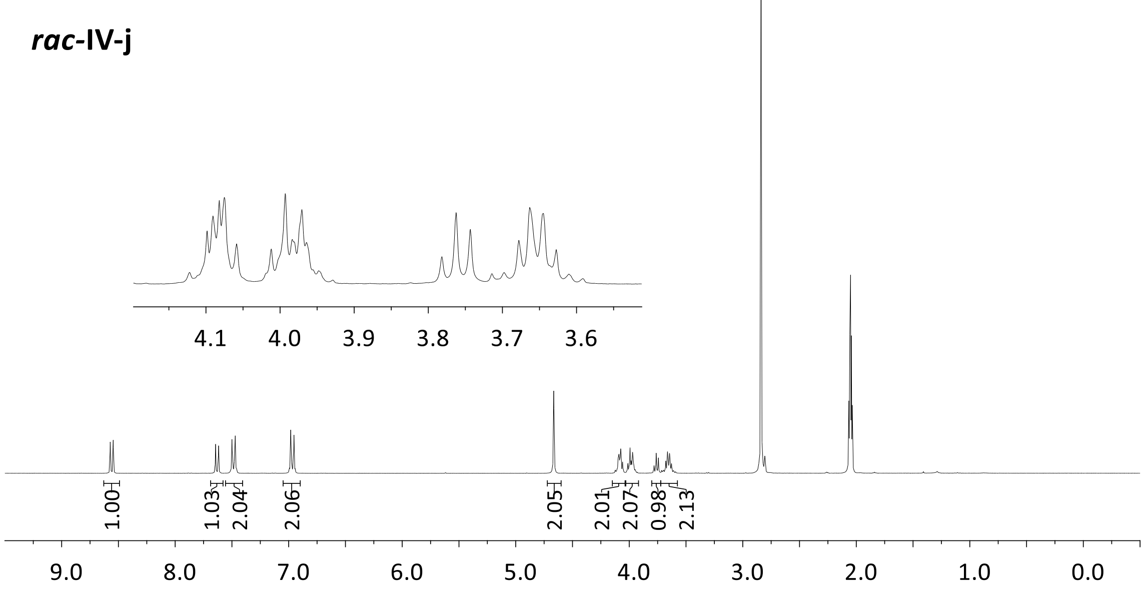
4.1
4.0
3.9
3.8
3.7
3.6
1.00
1.03
2.04
2.06
2.05
2.01
2.07
0.98
2.13
9.0
8.0
7.0
6.0
5.0
4.0
3.0
2.0
1.0
0.0

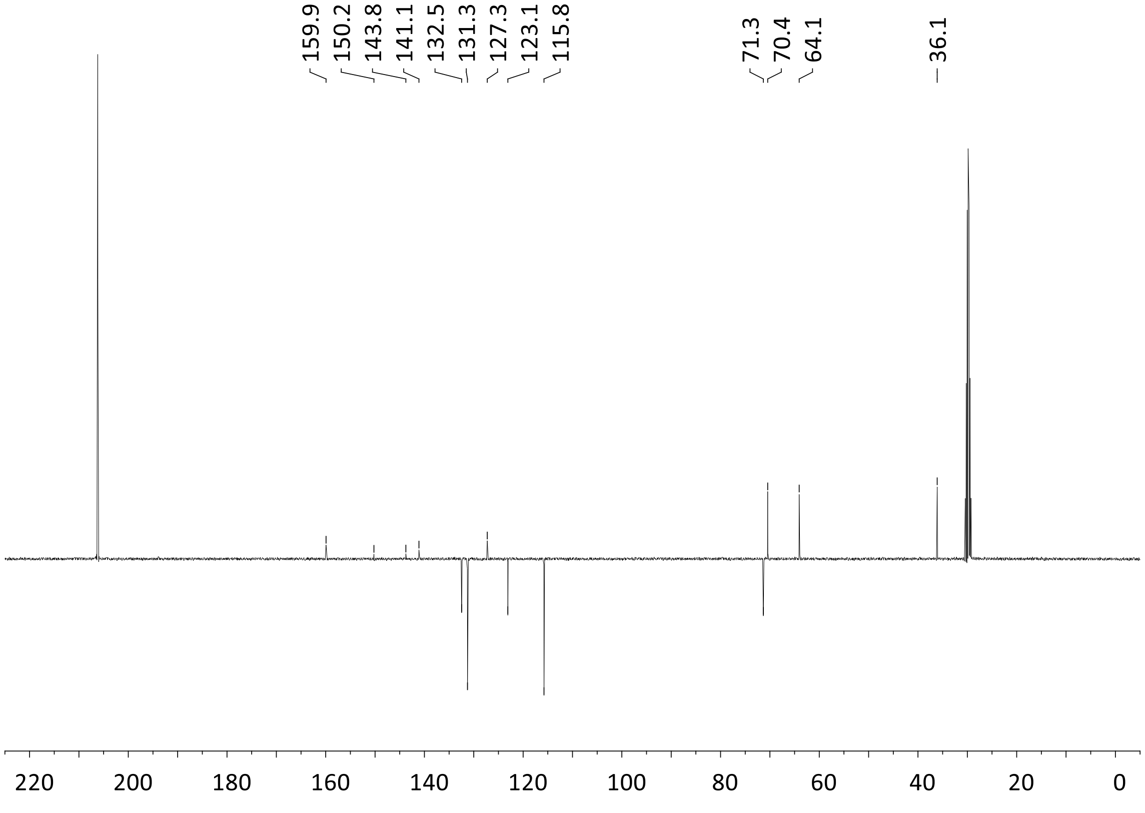
159.9
150.2
143.8
141.1
132.5
131.3
127.3
123.1
115.8
71.3
70.4
64.1
36.1
220
200
180
160
140
120
100
80
60
40
20
0

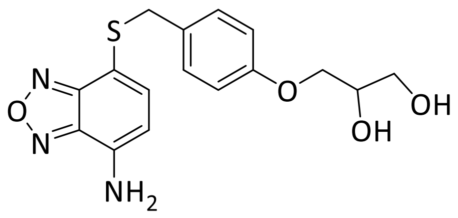
S
N
O
N
O
OH
OH
NH2

***rac*-IV-k**

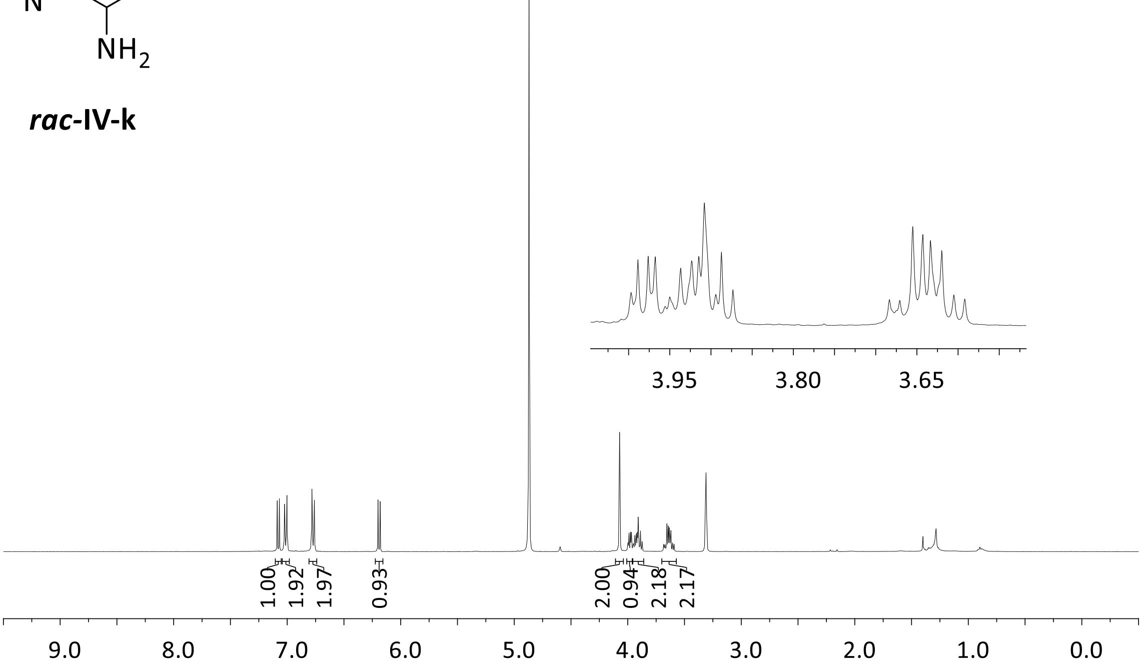
3.95
3.80
3.65
1.00
1.92
1.97
0.93
2.00
0.94
2.18
2.17
9.0
8.0
7.0
6.0
5.0
4.0
3.0
2.0
1.0
0.0

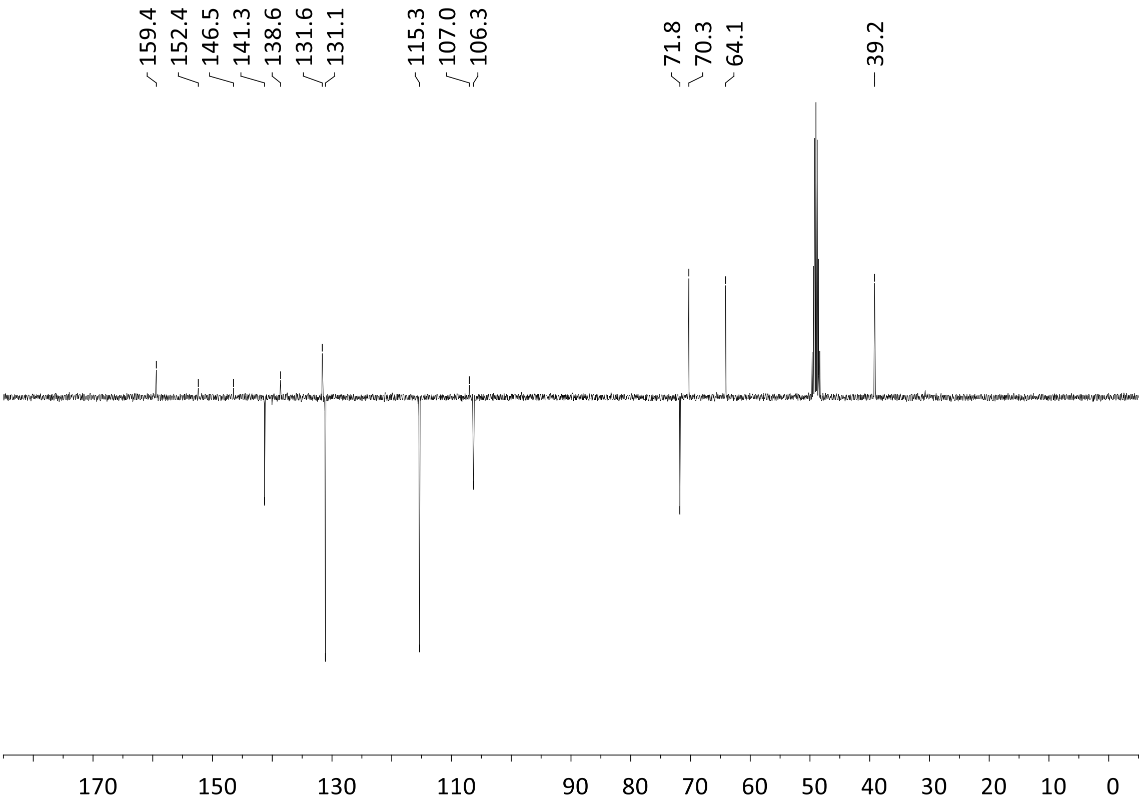
159.4
152.4
146.5
141.3
138.6
131.6
131.1
115.3
107.0
106.3
71.8
70.3
64.1
39.2
170
150
130
110
90
80
70
60
50
40
30
20
10
0

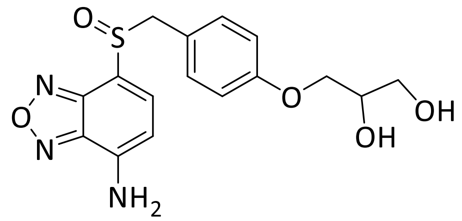

***rac*-IV-l**

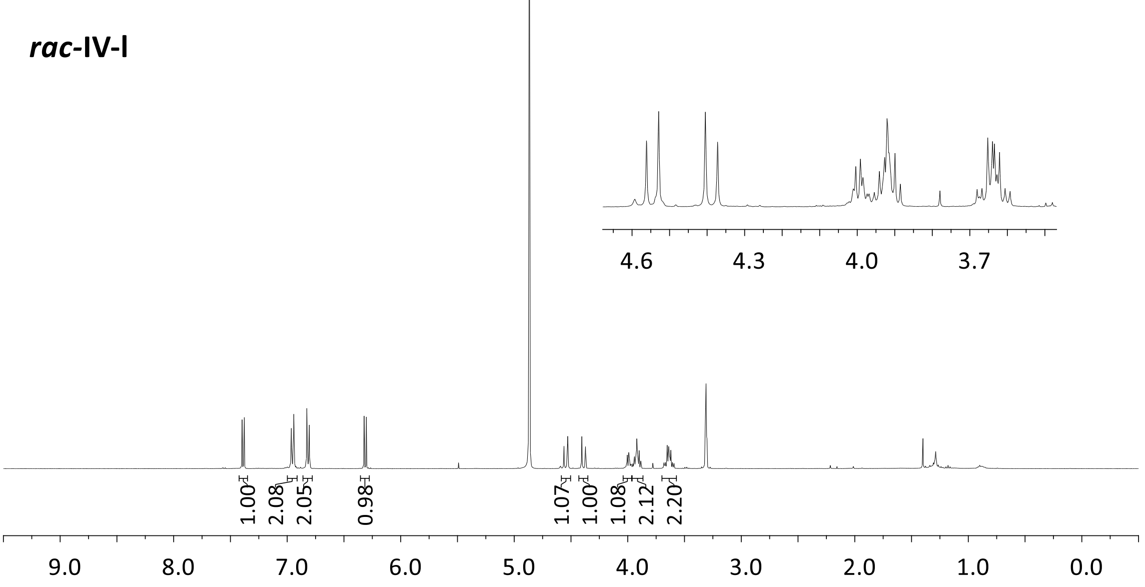
4.6
4.3
4.0
3.7
1.00
2.08
2.05
0.98
1.07
1.00
1.08
2.12
2.20
9.0
8.0
7.0
6.0
5.0
4.0
3.0
2.0
1.0
0.0

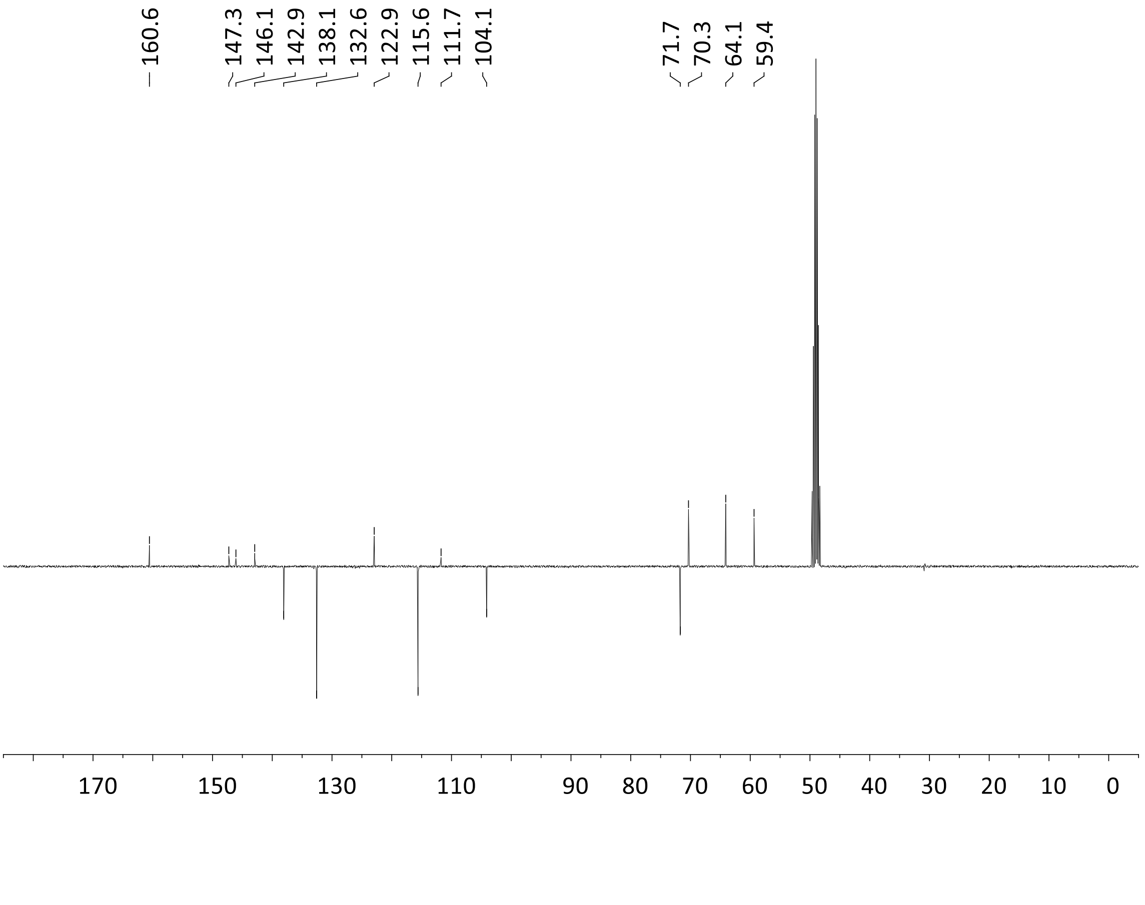
160.6
147.3
146.1
142.9
138.1
132.6
122.9
115.6
111.7
104.1
71.7
70.3
64.1
59.4
170
150
130
110
90
80
70
60
50
40
30
20
10
0
